# PuffAligner: a fast, efficient and accurate aligner based on the Pufferfish index

**DOI:** 10.1093/bioinformatics/btab408

**Published:** 2021-06-12

**Authors:** Fatemeh Almodaresi, Mohsen Zakeri, Rob Patro

**Affiliations:** Computer Science Department, University of Maryland, College Park, MD 20742, USA; Computer Science Department, University of Maryland, College Park, MD 20742, USA; Computer Science Department, University of Maryland, College Park, MD 20742, USA; Computer Science Department, University of Maryland, College Park, MD 20742, USA

## Abstract

**Motivation:**

Sequence alignment is one of the first steps in many modern genomic analyses, such as variant detection, transcript abundance estimation and metagenomic profiling. Unfortunately, it is often a computationally expensive procedure. As the quantity of data and wealth of different assays and applications continue to grow, the need for accurate and fast alignment tools that scale to large collections of reference sequences persists.

**Results:**

In this article, we introduce PuffAligner, a fast, accurate and versatile aligner built on top of the Pufferfish index. PuffAligner is able to produce highly sensitive alignments, similar to those of Bowtie2, but much more quickly. While exhibiting similar speed to the ultrafast STAR aligner, PuffAligner requires considerably less memory to construct its index and align reads. PuffAligner strikes a desirable balance with respect to the time, space and accuracy tradeoffs made by different alignment tools and provides a promising foundation on which to test new alignment ideas over large collections of sequences.

**Availability and implementation:**

All the data used for preparing the results of this paper can be found with 10.5281/zenodo.4902332. PuffAligner is a free and open-source software. It is implemented in C++14 and can be obtained from https://github.com/COMBINE-lab/pufferfish/tree/cigar-strings.

**Supplementary information:**

Supplementary data are available at *Bioinformatics* online.

## 1 Introduction

Since its introduction, next generation sequencing (NGS) has been widely used as a low-cost and accessible technology to produce high-throughput sequencing reads for many important biological assays.

The sequencing data that is generated in the form of short reads, drawn from longer molecular fragments, and finding the optimal alignments of these short reads to some reference is a necessary first step for many downstream biological analyses. The process of finding the segment on the reference that is most similar to the query read, and therefore most likely to be the source of the fragment from which the read was drawn, is known as read mapping or read alignment.

The main goal in read alignment is to find alignments of contiguous sub-string of the underlying reference that yields a minimum edit distance (or maximum alignment score) between the read and the reference sequence at the alignment position. If the reads are paired-end, characteristics other than the alignment score can be used to filter spurious alignment locations, such as orientation of each end of the alignment pair (forward or reverse) or distance between the alignments corresponding to reads that are ends of the same fragment.

Short-read aligners are a major workhorse of modern genomics. Given the importance of the alignment problem, a tremendous number of different tools have been developed to tackle this problem. Some widely used examples are BWA (Li and Durbin, 2009), Bowtie2 (Langmead and Salzberg, 2012), Hisat2 (Kim *et al.*, 2015, 2019) and STAR (Dobin *et al.*, 2013).

Existing alignment tools use a variety of indexing methods. Some tools, such as BWA, Bowtie2 and STAR use a full-text index over the reference sequences; BWA and Bowtie2 use variants of the FM-index, while STAR uses a suffix array.

A popular alternative approach to full-text indices is to instead, index sub-strings of length *k* (*k*-mers) from the reference sequence. Trading off index size for potential sensitivity, such indices can either index all of the *k*-mers present in the underlying reference, or some uniform or intelligently chosen sampling of *k*-mers. There are a large variety of *k*-mer-based aligners, including tools like the Subread aligner (Liao *et al.*, 2013), SHRiMP2 (David *et al.*, 2011), mrfast (Alkan *et al.*, 2009) and mrsfast (Hach *et al.*, 2010). To reduce the index size, one can choose to select specific *k*-mers based on a winnowing (or minimizer) scheme. This approach has been particularly common in tools designed for long-read sequence alignment like mashmap (Jain *et al.*, 2018) and minimap2 (Li, 2018).

Recently, a set of new indices for storing *k*-mers have been proposed based on graphs, specifically de Bruijn graphs (dBg). A de Bruijn graph is a graph over a set of distinct *k*-mers where each edge connects two neighboring *k*-mers that appear consequently in a reference sequence and therefore, overlap on ‘*k—*1’ bases. Kallisto (Bray *et al.*, 2016), deBGA (Liu *et al.*, 2016), BGreat (Limasset *et al.*, 2016), BrownieAligner (Heydari *et al.*, 2018) and Pufferfish (Almodaresi *et al.*, 2018) are some tools which use an index constructed over the de Bruijn graph built from the reference sequences. Cortex (Iqbal *et al.*, 2012), Vari (Muggli *et al.*, 2017), rainbowfish (Almodaresi *et al.*, 2017) and mantis (Pandey *et al.*, 2018) are also tools that use a colored compacted de Bruijn graph for building their index over a set of raw experiments. All these approaches cover a wide range of the possible design space, and different design decisions yield different performance tradeoffs.

Generally, the fastest aligners (like STAR) have very large memory requirements for indexing, and make some sacrifices in sensitivity to obtain their speed. On the other hand, the most sensitive aligners (like Bowtie2) have very moderate memory requirements but obtain their sensitivity at the cost of a higher runtime. Maintaining the balance between time and memory is especially more critical while aligning to a large set of references, like a large collection of microbial and viral genomes which may be used as an index in microbiome or metagenomic studies. As both the collection of reference genomes and the amount of sequencing data grows quickly, it is import for alignment tools to scale to such large collections of data and references with reasonable resource requirements while remaining fast and sensitive.

Based on the compact Pufferfish (Almodaresi *et al.*, 2018) index, we introduce a new aligner called PuffAligner, that we believe strikes an interesting and useful balance in this design space. PuffAligner is designed to be a highly sensitive alignment tool while, simultaneously, placing a premium on computational overhead. By using the colored compacted de Bruijn graph to factor out repeated sub-sequences in the reference, it is able to leverage the speed and cache friendliness of hash-table-based aligners while still controlling the growth in the size of the index; especially in the context of redundant reference sequences. Therefore, the index provides favorable scalability, in terms of index size, construction memory and time, compared to popular indexes such as Bowtie2 and STAR. By carefully exploring the alignment challenges that arise in different assays, including single-organism DNA-seq, RNA-seq alignment to the transcriptome, and metagenomic sequencing, we have engineered a versatile tool that strikes desirable balance between accuracy, memory requirements and speed. We compare PuffAligner to some other popular aligners and show how it navigates these different tradeoffs.

## 2 Materials and methods

PuffAligner is an aligner built on top of the Pufferfish indexing data structure. Pufferfish is a space-efficient and fast index for the colored compacted de Bruijn graph (ccdBg). A colored compacted de Bruijn graph is a graph whose vertices (strings) are the compacted non-branching paths of the underlying de Bruijn graph, with the restriction that each node also have the same color set (set of reference sequences in which it appears). The nodes in the colored compacted de Bruijn graph are referred to as unitigs. Each unitig can exist in multiple references and be mapped to a list of <reference ID, position orientation > tuples. The tuple describes the position and orientation with which the unitig subsequence appears in each reference. The basic query operation in the Pufferfish index is to query a *k*-mer from the input sequence against the index. Given this query, the Pufferfish index returns the unique position (and orientation) where this *k*-mer appears in the colored compacted de Bruijn graph (or a sentinel value if this *k*-mer does not occur). This match between the query and the graph can then be easily ‘unpacked’ into the implied list of matches with the underlying references by finding all of the places that the matched unitig appears in the reference sequences.

The main advantage of an aligner based on a compacted sequence graph, such as PuffAligner, offers compared to aligners based on linear full-text indices such as the FM-index, is the reduction of overhead related to computations for repeated subsequences within or across multiple references. This benefit results by performing the basic matching and mapping preprocessing steps on the graph itself, and annotating the results with respect to each reference, rather than performing these operations on all reference sequences individually. While *k*-mer query is the basic operation performed by Pufferfish index, we actually do not query for all the *k*-mer matches directly in PuffAligner. Instead, starting from *k*-mer *k*_0_, we extend the initial *k*-mer match into a unique maximal exact match [uni-MEMs, as introduced by (Liu *et al.* (2016)] which is the longest match of the read and the unitig containing *k*_0_. This uni-MEM is later used to construct a Maximal Exact Matches (MEM), which is a Maximal Exact Match shared between the read and the *reference*. We utilize the uniqueness property of unitigs to reduce the operational overhead of MEM extension **per each reference** to only **once for a unitig**. We expand a uni-MEM to different MEMs by assigning the reference information of the uni-MEM’s underlying unitig to the uni-MEM. Using this approach, a uni-MEM can be a part of multiple MEMs, just as a unitig can be a part of multiple references. During MEM assembly, we merge overlapping consequent uni-MEMs that are only separated because of small branched unitigs. In this way, we guarantee the accuracy, performance of later mapping steps, and obtain similar behavior that is expected from dealing with chains of MEMs in more traditional alignment approaches. Finally, rather than fully aligning each query sequence to the anchored position on the reference, only the sub-sequences from the query that are not part of the MEMs (exact matches) appearing within the current high-scoring MEM chain are aligned to the reference; we call this procedure between-MEM alignment. Each of these steps are explained in detail in the following sections.

### 2.1 Exact matching in the Pufferfish index

The Pufferfish index provides PuffAligner with an efficient index for *k*-mer lookup within a list of references such as a collection of transcripts, genes or genomes. Specifically, the core components of the index are (i) a minimal perfect hash function (MPHF), (ii) a unitig sequence vector, (iii) a unitig -to-reference table and (iv) a vector storing the position associated with each *k*-mer in the unitig sequence vector. The unitig sequence vector contains all the unitigs in the ccdBg. In the Pufferfish index, a unitig is a monochromatic path in the colored compacted de Bruijn graph, where a color corresponds to a *set* of reference identifiers. Hence, each unitig is a substring of some set of reference sequences. Each unitig is annotated with the list of references that contain the unitig’s sequence. In this design, a unitig can belong to multiple references and a reference can contain multiple unitigs.

The Pufferfish index admits efficient exact search for *k*-mers, as well as longer matches that are unique in both the query string and colored compacted de Bruijn graph. These matches, called uni-MEM, were originally defined in deBGA (Liu *et al.*, 2016). A uni-MEM is a Maximal Exact Match (MEM) between the query sequence and a unitig. Using the combination of the MPHF and the position vector, a *k*-mer is mapped to a unitig in the unitig sequence vector. The *k*-mer is then extended to a uni-MEM via a linear scan of the query sequence and the unitig sequence vector. Each uni-MEM can appear in multiple different references, and since uni-MEMs must be completely contained within a unitig, it is possible for multiple uni-MEMs to be directly adjacent on both the query and some references where the unitig appears.

*uni-MEM collection*: The first step in read alignment is to collect exact matches shared between the query (single-end or paired-end reads) and the reference. In PuffAligner, this is accomplished by collecting the set of uni-MEMs that co-occur between the query and reference. PuffAligner starts processing the read from the left-end and looks up each *k*-mer that is encountered until a match to the index is found. Once a match is discovered, it is extended in both query and the reference until one of these termination conditions occur: (i) a mismatch is encountered, (ii) the end of the query is reached or (iii) the end of the unitig is reached. This process results in a uni-MEM match shared between the query and reference. uni-MEMs where extension is terminated as a result of reaching the end of a unitig must later be examined and potentially ‘collpased’ together to form MEMs with respect to the references on which they appear. If the uni-MEM extension is not terminated as a result of reaching the end of the query, then the position in the read is incremented by a small value and the same procedure is repeated for the next *k*-mer on the read. This process continues until either the uni-MEM extension terminates because the end of the query is reached, or because the last *k*-mer of the query is searched in the index. Here, we recall an important property of uni-MEM extension that is different from e.g. MEM extension or maximum mappable prefix (MMP) extension (Dobin *et al.*, 2013). Due to the definition of the ccdBg, it is guaranteed that any *k*-mer appearing within a uni-MEM cannot appear in any other unitig in the ccdBg. Thus, extending *k*-mers to maximal uni-MEMs is safe with respect to greedy extension, as such extension will never cause missing a *k*-mer that would lead to another distinct uni-MEM shared between the query and reference. The concept of safe extension of kmer matches was introduced in (Sarkar *et al.*, 2018).

*Filtering highly repetitive uni-MEMs*: In order to avoid expending computation on performing the subsequent steps on regions of reads mapping to highly repeated regions of the reference, any uni-MEM that appears more than a user-defined number of times in the reference is discarded. In this manuscript, we use the threshold of 1000. This filter has a strong impact on the performance, since, even if one *k*-mer from the read maps to a highly repetitive region of the reference, the following expensive steps of the alignment procedure should be performed for every mapping position of the uni-MEM to find the right alignment for the read, while the less repetitive uni-MEMs also map to the true origin of the read on the reference as well. The drawback of this filter is that for a very small fraction of the reads which are truly originating from a highly repetitive region, all of the matched uni-MEMs will be filtered out and no hit remains for aligning the read. However, we find that in the case of aligning paired-end reads, usually one end of the read maps to a non-repetitive region, then, the alignment of the other end can be recovered using orphan recovery (explained in Section 2.4). Furthermore, we also provide a flag *–allowHighMultiMappers* that mitigates the effect of this filter for a slight tradeoff on the alignment performance.

*uni-MEM compaction*: For paired-end reads, PuffAligner aligns each end of the read pairs individually. For each end, all the uni-MEMs are sorted on the basis of their positions on the reference. First the uni-MEMs are ‘unpacked’ to their corresponding unitig’s reference list and assigned a reference position by adding the unitig’s relative position in the reference to the uni-MEM’s position in the unitig. After mapping the uni-MEMs to the references, it is now possible to construct the <read end, reference > MEMs. Each MEM is defined as either a single uni-MEM or a combination of those consecutive uni-MEMs that have an overlap of *k—*1 on both the query and reference (which is equal to unitigs’ overlap).

The compactable uni-MEMs result from terminating the extension process due to reaching the end of a unitig. After the compaction of uni-MEMs, there is a list of MEMs which are shared sequences between the query and a set of reference positions, that are sorted based on the reference positions.

Generally, a MEM is calculated for each pair of read and reference. This means that, in the case of highly similar references, the process of finding the same MEM of the read has to be repeated for each reference. Through the indirect construction of MEMs from uni-MEMs we gain a performance boost by extending the *k*-mer once per read and simply using the extended subsequence in MEMs of the read to difference references. However, the shorter the unitigs, the shorter the uni-MEMs. Therefore, the more *k*-mers per read need to be queried. This will affect the performance of MEM construction which, in the worst-case scenario, is required to query for each *k*-mer in the read. However, the *k*-mer queries still only happen once per each read and not once per each pair of read and reference.

### 2.2 Finding promising MEM chains

**Fig. 1.**
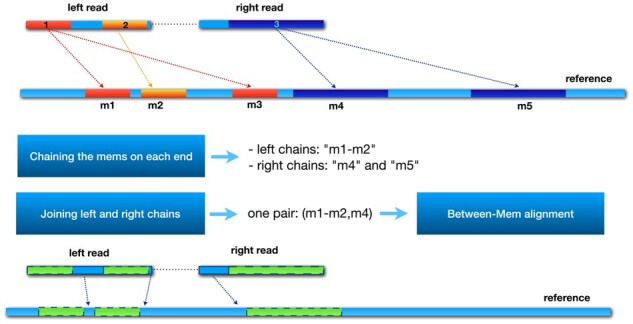
This figure shows the main steps of chaining and between-MEM alignment in the PuffAligner procedure via an example. In this example, m1, m2 and m3 are the projected MEMs from the left end of the read to the reference and m4 and m5 are the projected MEMs from the right end of the read. In the first step, the chaining algorithm chooses the best chain of MEMs that provide the highest coverage score for each end of the read, that is the m1-m2 chain for the left end and two single MEM chain for the right end. Then, the selected chains from each end are joined together to find the concordant pairs of chains, that is the (m1-m2, m4) pair for this read as m5 is too far from m1-m2. Then, the chain from each end will go through to the next step, between-MEM alignment. For the green (dashed border) areas (MEMs) no alignment is recalculated as they are exact matches. Only the un-matched blue (solid border) parts of the reads (those nucleotides not occurring within a MEM) are aligned using a modified version of KSW2

Via the procedures described above, we have enumerated all of the possible MEMs of length at least *k* shared between a read and the reference sequences. As shown in Figure 1, having all the MEMs from a read to each target reference, the goal of this step is to find promising chains of MEMs that cover the most unique bases in the read in a concordant fashion and that can potentially lead to a high quality alignment.

To accomplish this, we adopt the dynamic programming approach used in minimap2 (Li, 2018) for finding co-linear chains of MEMs that are likely candidates to support high-scoring read alignments. As mentioned in minimap2, all the MEMs from a read *r* to the reference *t*, are sorted by the ending position of the MEMs on the reference. Then, this algorithm computes a score for each set of MEMs based on the number of unique covered bases in the read, the coverage score is also penalized by the length of the gaps, both in the read and reference sequence, between each consecutive pair of MEMs.

In PuffAligner, if the distance between two MEMs, *m*_1_ and *m*_2_, on the read and the reference is *d_r_* and *d_t_* respectively, these two MEMs should not be chained together if $|d_{r}-d_{t}|>C$, where *C* is the maximum allowed gap. So, the penalization term, the *β* value in (Li, 2018), in the coverage score computation is modified accordingly to prevent pairing of such MEMs.

Also, unlike what is done in minimap2 (Li, 2018), rather than considering together the MEMs that are discovered on both ends of a paired-end read, we consider the chaining for each end of the read separately. This is done in order to make it easier to enforce the orientation consistency of the individual chains. Specifically, the chaining algorithm that is presented in (Li, 2018) introduces a transition in the recursion that can be used to switch between the MEMs that are part of one read and those that are part of the other. However, such switching makes it difficult to enforce the orientation consistency of the chains that are being built for each end of the read. One solution to this problem is to add another dimension to the dynamic programming table, encoding if one has already switched from the MEMs of one read end to the other, and the recurrence can be modified to allow only one switch from the one read end to the other, allowing enforcement of orientation consistency. However, we found that, in practice, simply chaining the read ends separately led to better performance.

Finally, we also adopt the heuristic proposed by minimap2 (Li, 2018) when calculating the highest scoring chains. That is, when a MEM is added to the end of an existing chain, it is unlikely that a higher score for a chain containing this MEM will be obtained by adding it to a preceding chain. Thus, we consider only a small fixed number of rounds (by default 2) of preceding chains once we have found the first chain to which we can add the current MEM.

The chaining algorithm described above finds the best chains of MEMs shared between the read *r* and the reference *t* in orientation *o*. A chain is accepted if its score is greater than a configurable fraction, which we call the *consensusFraction*, times the length of the read *r*. Throughout all the experiments in this manuscript the *consensusFraction* is set to 0.65. If a chain passes the consensus fraction threshold, we call it a *valid* chain. In addition, rather than keeping all valid chains, we also filter highly suboptimal chains with respect to the highest scoring chain *per-reference*. All valid chains shared between *r* and *t* are sorted by their scores, and chains having scores within 10% of the highest scoring chain for reference *t* are selected as potential mappings of the read *r* to the reference *t*. While these filters are essential for improving the throughput of the algorithm in finding the right alignment, they are carefully selected to have very little effect on the sensitivity of PuffAligner. For all the experiments in this manuscript, the same default settings of these parameters are used if not mentioned otherwise.

### 2.3 Computing base-to-base alignments between MEMs

After finding the high-scoring MEM chains for each reference sequence, a base-to-base alignment of the read to each of the candidate reference sequences is computed. Each selected chain implies a position on the reference sequence where the read might exhibit a high quality alignment. Thus, we can attempt to compute an optimal alignment of the read to the reference at this implied position, potentially allowing a small bit of padding on each side of the read. This approach utilizes the positional information provided by the MEM chains. However, the starting position of the alignments is not the only piece of information embedded in the chains. Rather each chain of MEMs consists of sub-sequences of the read (of size at least *k*, though often longer) which match exactly to the reference. While the optimal alignment of the read to the reference at the position being considered is not *guaranteed* to contain these exact matches as alignments of the corresponding substrings, this is almost always the case in practice.

In PuffAligner, we aim to exploit the information from the long matches to accelerate the computation of the alignments. In fact, since only chains with relatively high coverage score are selected, a large portion of the read sequences are typically already matched to the positions in the reference with which they will be matched in the final optimal alignment. For instance, in Figure 1, for the final chains selected on the reference sequence, it is already known for the red and orange sub-sequences (areas 1 and 2) on the left end of the read precisely where they should align to the reference. Likewise this is the case for the purple sub-sequence (area 3) on the right read. The unmapped regions of the reads are either bordered by the exact matches on both sides, or they occur at the either ends of the read sequence. In fact, other aligners such as STAR (Dobin *et al.*, 2013), BWA-MEM (Li and Durbin, 2009) and minimap2 (Li, 2018) employ the same strategy to assume that the exact matches, found during the query time, are part of the final alignments to be reported. PuffAligner skips aligning the whole read sequence by considering the exact matches of the MEMs to be part of the alignment solution. As a result, it is only required to compute the alignment of the small unmapped regions, which reduces the computation burden of the alignments.

When applying such an approach, two different types of alignment problems are introduced, which we call bounded sub-sequence alignment and ending sub-sequence alignment. For bounded sub-sequence alignment, we need to *globally* align some interval *i_r_* of the read to an interval *i_t_* of the reference. If *i_r_* and *i_t_* are of different lengths, the alignment solution will necessarily include insertions or deletions. If *i_r_* and *i_t_* are of the same length, then the optimal global alignment between them may or may not include indels. For each such bounded sub-sequence alignment, we determine the optimal alignment of *i_r_* to *i_t_* by computing a global pair-wise alignment between the intervals, and stitching the resulting alignment together with the exact matches that bound these regions.

Gaps at the beginning or the end of the read are symmetric cases and so we describe, without loss of generality, the case where there is an unaligned interval of the read after the last MEM shared between the read and the reference. In this case, we need to solve the ending sub-sequence alignment problem. Here, the unaligned interval of the read consists of the substring spanning from the last nucleotide of the terminal MEM in the chain, up through the last nucleotide of the read. There is not a clearly defined interval on the reference sequence. While the left end of the relevant reference interval is defined by the last reference nucleotide that is part of the bounding MEM, the right end of the reference interval should be determined by actually solving an extension or ‘end-free’ alignment problem. We address this by performing extension alignment of the unaligned interval of the read to an interval of the reference that begins on the reference at the end of the terminal MEM, and extends for the length of the unaligned query interval plus the length of some problem-dependent buffer (which is determined by the maximum length difference between the read and reference intervals that would still admit an alignment within the acceptable score threshold).

PuffAligner uses KSW2 (Li, 2018; Suzuki and Kasahara, 2018) for computing the alignments of the gaps between the MEMs and for aligning the ending sequences. KSW2 exposes a number of alignment modes such as global and extension alignments. For aligning the bounded regions, KSW2 alignment in the global mode is performed, and for the gaps at the beginning or end of reads, PuffAligner uses the extension mode to find the best possible alignment of that region. For increasing the efficiency of alignment computation we also employ some specific techniques in PuffAligner which are explained in Supplementary Section S1.

### 2.4 Joining mappings for read ends

Finally, once alignments have been computed for the individual ends of a read, they must be paired together to produce valid alignments for the entire fragment. At this point in the process, on each reference sequence, there are a number of locations where the left end of each read or the right end of each read, or both, are mapped to the reference. For the purpose of determining which mappings will be reported as a valid pair, the mappings are joined together only if they occur on opposite strands of the reference, and if they are within a maximum allowed fragment length. There are two different types of paired-end alignments that can be reported by PuffAligner; concordant and discordant. If PuffAligner is disallowed from reporting discordant alignments, then the mapping orientation of the left and right end should agree with the library preparation protocols of the reads. PuffAligner first tries to find concordant mapping pairs on a reference sequence, and if no concordant mapping is discovered and the tool is being run in a mode where discordant mappings are allowed, then PuffAligner reports pairs that map discordantly. Here, discordant pairs may be pairs that do not, for example, obey the requirement of originating from opposite strands. While this is not expected to happen frequently, it may occur if there has been an inversion in the sequenced genome with respect to the reference.

## 3 Results

For measuring the performance of PuffAligner and comparing it to other aligners, we have designed a series of experiments using both simulated and experimental data from different sequencing assays. We compare PuffAligner with Bowtie2 (Langmead and Salzberg, 2012), STAR (Dobin *et al.*, 2013) and deBGA (Liu *et al.*, 2016). Bowtie2 is a popular, sensitive and accurate aligner with the benefit of having very modest memory requirements. STAR requires a much larger amount of memory, but is much faster than Bowtie2 and can also perform ‘spliced alignment’ against a reference (which PuffAligner, Bowtie2 and deBGA currently do not allow). deBGA, is most-related tool to PuffAligner conceptually, as it is an aligner with a colored compacted de Bruijn graph-based index that is focused on exploiting redundancy in the reference sequence.

We use different metrics to assess both the performance and accuracy of each method on a variety of types of sequencing samples. These experiments are designed to cover a variety of different use-cases for an aligner, spanning the gamut from situations where most alignments are expected to be unique (DNA-seq), to situations where each fragment is expected to align to many loci with similar quality (RNA-seq and metagenomic sequencing), and spanning the range of index sizes from small transcriptomes to large collections of genomes.

PuffAligner’s underlying index, Pufferfish supports two index variants, ‘dense’, and ‘sparse’ (Dense is the default index and sparse is activated using the ‘-s’ option in the index build step). The ‘sparse’ version trades off lookup time for index size, and provides a smaller index in which *k*-mer lookups take longer. However, in the alignment process described here, since the steps after the initial lookup are the main bottleneck in alignment time (e.g. compacting uni-MEMs into MEMs, chaining MEMs and finally performing between-MEM alignment), we recommend the sparse Pufferfish index to be the choice. This is the index version we have used for all experiments in this manuscript.

First, we show PuffAligner exhibits similar accuracy for aligning DNA-seq reads to Bowtie2, but it is considerably faster. In the case of experimental reads, since the true origin of the read is unknown, we use measures such as mapping rate and concordance of alignments to compare the methods. Furthermore, in Supplementary Section S7 we evaluate the accuracy of aligners by aligning simulated DNA-seq reads that include variation (single-nucleotide variants and small indels with respect to the reference). For aligning RNA-seq reads, we compare the impact of alignments produced by each aligner on downstream analysis such as abundance estimation. Finally, we show PuffAligner is very efficient for aligning metagenomic samples where there is a high degree of shared sequence among the reference genomes being indexed. We also illustrate that using alignments produced by PuffAligner yields the highest accuracy for abundance estimation of metagenomic samples.

### 3.1 Alignment of whole genome sequencing reads

First, we evaluate the performance of PuffAligner with a whole genome sequencing (WGS) sample from the 1000 Genomes project (Consortium *et al.*, 2015). We downloaded the ERR013103 reads from sample HG00190, which is a low-coverage sample from a Finnish male, sequenced in Finland (https://www.internationalgenome.org/data-portal/sample/HG00190). There are 18 297 585 paired-end reads, each of length 108 nucleotides in this sample. Using fastp (Chen *et al.*, 2018), we remove low quality ends and adapter sequences from these reads. After trimming, there are 15 404 412 reads remaining in the sample. Indices for each of the tools are built over all DNA chromosomes of the human genome [GRCh38 (Schneider *et al.*, 2017)] which is obtained from gencode release v33 (Frankish *et al.*, 2019) (https://www.gencodegenes.org/human/release_33.html). All the tool, if possible, are run in the concordant-mode in this experiment.

The alignment rate, run-time memory usage and running time for all the aligners are presented in Table 1. The reason that deBGA has the highest mapping rate in Table 1 compared to other tools is that it is local alignments for the reads that are not alignable end-to-end under the scoring parameters for the other tools. Bowtie2 and PuffAligner are both able to find end-to-end alignments for about ∼95% of the reads. STAR and PuffAligner are the fastest tools, with STAR being somewhat faster than PuffAligner. On the other hand, PuffAligner is able to align more reads than STAR, while requiring less than half as much memory. The memory usage of Bowtie2 is the smallest, since Bowtie2’s index does not contain a hash table. However, this comes at the cost of having the longest running time compared to other methods. Overall, PuffAligner benefits from the fast query of hash-based indices while its run-time memory usage, which is mostly dominated by the size of the index, is significantly smaller than other hash-based aligners. Although deBGA’s index is based on the de Bruijn graphs, similar to the Pufferfish index, the particular encoding for it is not as space-efficient as that of Pufferfish.

**Table 1.** The performance of different tools for aligning experimental DNA-seq reads

Aligner	Mapping-rate (%)	Time (mm:ss)	Memory (GB)
PuffAligner	95.58	6:14	13.09
deBGA	99.75	10:46	41.04
STAR	93.88	4:29	30.36
Bowtie2	95.44	16:15	3.50

*Note*: The time reports are benchmarked after warming up the system cache so that the influence of index loading time is mitigated.

To look more closely how the mappings between the tools differ, we investigate the agreement of the reads which are mapped by Bowtie2, STAR and PuffAligner. We are only comparing these three methods which perform end-to-end alignment in this plot, since outliers from the local alignments computed by deBGA would otherwise dominate the plot. Majority of the reads (∼14.2M reads) are mapped by all three aligners. The next largest set (∼400K reads) represents the reads which are only mapped by Bowtie2 and PuffAligner. All the other sets are much smaller compared to the first two sets. This fact illustrates that the highest agreement in the aligners is between Bowtie2 and PuffAligner. We have also visualized the results in an upset plot in Supplementary Figure S1 using the UpsetR library (Conway *et al.*, 2017).

Exploring a series of individual reads from the smaller sets in the upset plot, suggests that some of these differences happen as a result of small differences in the scoring configuration, while some result from different search heuristics adopted by the different tools. Supplementary Figure S2 shows the coherence between the alignments reported by the tools by also including the exact location to which the reads are aligned in the reference.

### 3.2 Transcript abundance estimation from RN-seq reads

Mapping sequencing reads to target transcriptomes is the initial step in many pipelines for reference-based transcript abundance estimation. While lightweight mapping approaches (Bray *et al.*, 2016; Patro *et al.*, 2017) greatly speed-up abundance estimation by, in part, eliding the computation of full alignment between reads and transcripts, there is evidence that alignments still yield the most accurate abundance estimates by providing increased sensitivity and avoiding spurious mappings (Sarkar *et al.*, 2018; Srivastava *et al.*, 2020; Vuong et al., 2018). Thus, the continued development of efficient methods for producing accurate transcriptome alignments of RNA-seq reads remains a topic of interest.

In Supplementary Section S6, we compare the effect of alignments produced by each tool on the accuracy of RNA-seq abundance estimation. We find that RNA-seq quantification based on alignments produced by PuffAligner, STAR and Bowtie2 reaches the same level of accuracy. However, PuffAligner is the fastest aligner, being at least 1.5× faster than STAR and 25× faster than Bowtie2 while the memory usage by PuffAligner is only 2× larger than memory used by Bowtie2 and 3.4× smaller than STAR (full details in Section 6).

### 3.3 Alignment to a collection of microorganisms—simulated short reads

One main property of metagenomic samples is that they contain reads from a variety of genomes. Some of these genomes are highly similar and some are not even assembled yet—and hence unknown. To demonstrate the performance and accuracy of PuffAligner for metagenomic samples, we design two different experiments. The first which we call ‘Single-strain’ experiment is designed to specifically evaluate issues related to the similarity challenge and the second, the ‘bulk’ experiment, evaluates aligners in the presence of a high variety of species in the sample in addition to the high similarity of references. We discuss the results of the ‘bulk’ experiment which is a more comprehensive one, in the following paragraphs. The ‘single-strain’ experiment is described in Supplementary Section S8.

We construct the indices of PuffAligner, Bowtie2, STAR and deBGA on a random set of 4000 complete bacterial genomes downloaded from the NCBI microbial database. Supplementary Table S6 shows the time and memory required for constructing each of the indices, in addition to the size of the final index on disk. Overall, PuffAligner and Bowtie2 show a similar trend in time and memory requirements, while STAR and deBGA require an order of magnitude more memory. In terms of the final index size, Bowtie2 has the smallest index, PuffAligner has the second-smallest, and STAR has the largest.

We select three Illumina WGS samples with accession IDs SRR10948222 (Fisher, 2020), SRR11283975 and SRR11496426, the details of which are explained in Supplementary Table S4 and simulate ∼50M reads for each through the process explained in Supplementary Section 9.1.

The assessment of ‘accuracy’ directly from the aligned reads is not a trivial task. Due to the high rate of multi-mapping in these simulated samples, and due to the fact that multiple references can produce alignments of the same quality as the ‘true’ origin of the read, we calculate the accuracy by comparing the true and estimated abundances using a quantification tool (in this case, Salmon) rather than by comparing the read alignments directly. In Table 2 the accuracy metrics are calculated over the abundance estimations obtained using the alignments produced by running the aligners in the different modes specified. The list of metrics for metagenomic expression evaluations are *Spearman Correlation*, *Mean Absolute Relative Difference (MARD)*, *Mean Absolute Error (MAE)* and *Mean Squared Log Error (MSLE)*. The metrics have been chosen to be similar to previous work such as in Bracken (Lu *et al.*, 2017) and Karp (Reppell and Novembre, 2018). The definition of each of these metrics is provided in Supplementary Equation S2 in Supplementary Material.

**Table 2.** Accuracy of abundance estimation with Salmon using alignments reported by each aligner for the mock metagenomic sample simulated from SRR10948222

Alignment mode	Tool	Spearman	MARD	MAE	MSLE
Primary	PuffAligner	0.69	0.028	**1.39**	0.08
	Bowtie2	0.58	0.053	2.91	0.15
	STAR	**0.727**	**0.023**	1.493	**0.05**
	deBGA	0.28	0.616	656.08	6.53
Up to 20	PuffAligner	0.9	0.006	0.40	0.006
	Bowtie2	0.85	0.01	**0.22**	0.012
	STAR	**0.929**	**0.004**	0.303	**0.002**
	deBGA	0.28	0.573	637.60	5.65
Up to 200	PuffAligner	0.97	0.002	0.36	0.001
	Bowtie2	**0.99**	**0.001**	**0.19**	**0.00**
	STAR	0.929	0.004	0.299	0.002
	deBGA	0.28	0.571	637.83	5.55
Best strata	PuffAligner	**0.97**	**0.002**	0.36	**0.001**
	STAR	0.929	0.004	**0.3**	0.002

*Note*: All aligners are run in three main modes; allowing only one best alignment with ties broken randomly (Primary), up to 20 alignments reported per read and up to 200 alignments reported per read. PuffAligner and STAR support a fourth mode that allows reporting all equally best alignments (bestStrata). This option improves the performance while maintaining the accuracy of the results. Best result in each metric is highlighted in bold.

This experiment leads to three main observations. First, regardless of the alignment mode, quantifications derived from the deBGA alignments seem to lead to systematic underestimation of abundance. However, PuffAligner, STAR and Bowtie2, show very similar behavior with respect to accuracy. STAR is the best in primary mode as well as when allowing 20 alignments, closely followed by PuffAligner. When allowing up to 200 alignments per read, Bowtie2 tends to yield the most accurate abundances, again with PuffAligner being the close runner-up. These results demonstrate that PuffAligner is a reliable alignment tool showing a stable pattern of being comparable to the best aligner under all the scenarios tested. That is, the good performance of PuffAligner is robust across a variety of different parameter settings.

Moreover, due to the nature of the metagenomic data—the high degree of ambiguity and multi-mapping—we expect to see improvement in the accuracy metrics as more alignments are reported per read, as this leads to a higher recall. While STAR’s accuracy changes only slightly from 20 alignments to 200 alignments (only improving MAE) the results for PuffAligner and Bowtie2 improve considerably when allowing more alignments per read. However, this higher accuracy comes in the cost of alignment time for Bowtie2 as shown in Figure 3. The difference becomes especially evident when allowing up to 200 alignments per read, where PuffAligner is 4 times faster than Bowtie2. In addition, in experimental data, many of the alignments reported do not necessarily have high quality, and only appear in the output as one of the 200 alignments for the read. In fact, we note the similar accuracy achieved by PuffAligner in *bestStrata* mode compared to when we allow up to 200 alignments per read. This observation is also consistent across the other two simulated samples in Supplementary Table S5, in those cases with PuffAligner being the most accurate aligner in different modes for both samples.

**Fig. 2.**
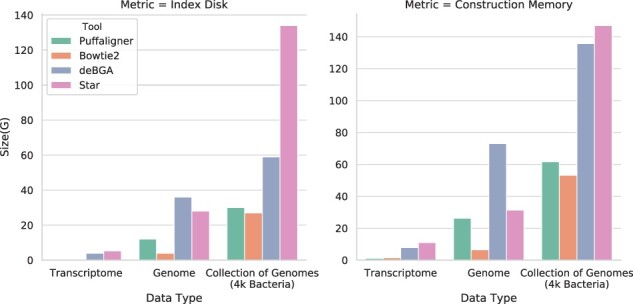
Time performance for aligning a mock experiment simulated from bulk read sample SRR10948222. The dashed area shows fraction of the time spent purely on aligning reads and the rest is the time required for index loading. PuffAligner is the fastest tool, yet most of its time is still dedicated to loading the index. The alignment for Bowtie2 increases when asking for more alignments per read while the other tools show a constant alignment time scaling over number of reads

**Fig. 3.**
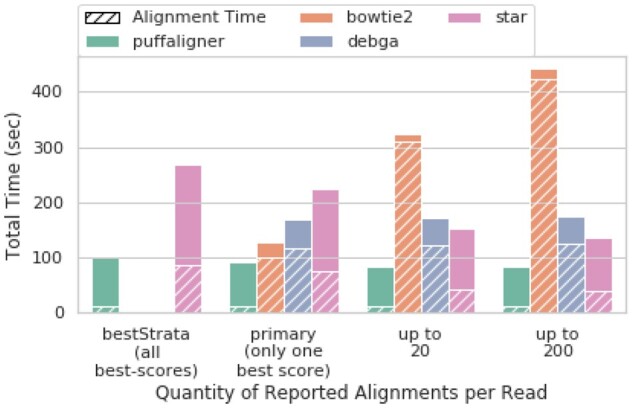
Scalability of different tools over the final index disk and c onstruction memory for three different datasets, human transcriptome (gencode version 33), human genome (GRCh38 primary assembly) and collection of genomes (4000 random bacterial complete genomes)

Overall, these results along with other similar experiments in Supplementary Section S8 and Supplementary Table S5 indicate that PuffAligner is a sensitive and fast aligner. Specifically PuffAligner exhibits similar accuracy (and is sometimes more accurate) as well-known aligners like Bowtie2 and STAR. On these data, it exhibits memory requirements close to those of the memory-frugal Bowtie2, while being much faster. Figure 3 shows that PuffAligner has the lowest running time, even when the number of allowed alignments per read increases. In Supplementary Section S11, we explain the reasons and advantages behind using PuffAligner as opposed to a light-weight pipeline like Kraken2 + Bracken for metagenomic analyses with examples from real metagenomic experiments.

### 3.4 Scalability

Figure 2 and Supplementary Figure S7 represent how the construction memory and index size of each tool scales over different types of sequences. The trend shows the effect of database size as well as redundancy and sequence similarity on the scalability of each of the tools. Tools such as PuffAligner and deBGA, which build a de Bruijn graph-based index on the input sequence, specifically compress similar sequences into unitigs and therefore scale well for databases with high redundancy such as collections of related microbial genomes. As the figure shows, the size of the PuffAligner index increases relatively less compared to that of Bowtie2 and STAR as we add more (specifically, repetitive) sequences so that, although the PuffAligner index is almost three times larger than the Bowtie2 index for the human genome, PuffAligner’s index is similar in size to that of Bowtie2 over the references for the metagenomic experiments. It is worth mentioning that Bowtie2 requires a switch from a 32-bit index to a 64-index as the total count of the input bases increases, which is another reason why the size is growing super-linearly. To better evaluate how the different indices grow as a function of the size of the reference database and similarity of the sequences that constitute the database, we designed a small-scale experiment where we compare the scalability of Bowtie2 and PuffAligner when increasing the size of the database under two different scenarios. In one case, we index a collection of increasing size of highly similar genomes, and in the other we index a collection of increasing size of genomes from distinct species (with much less sequence similarity). In Supplementary Section 10, we demonstrate the scalability advantage of PuffAligner for indexing highly redundant and similar reference collections.

## 4 Discussion and conclusion

In this article, we introduce PuffAligner, an aligner suitable for the contiguous alignment of short-read sequencing data. We demonstrate its use in aligning DNA-seq reads to the genome of a single species, aligning RNA-seq reads to the transcriptome, and aligning DNA-seq reads from metagenomic samples to a large collection of references. It is built on top of the Pufferfish index, which constructs a colored compacted de Bruijn graph using the input reference sequences. PuffAligner begins read alignment by collecting unique maximal exact matches, querying *k*-mers from the read in the Pufferfish index. The aligner then chains together the collected uni-MEMs using a dynamic programming approach, choosing the chains with the highest coverage as potential alignment positions for the reads. Finally, PuffAligner is able to efficiently compute alignment, exploiting information from long matches in the chains and making use of an alignment cache to avoid redundant work.

We compared the accuracy and efficiency of PuffAligner against two widely used alignment tools, Bowtie2 and STAR, that perform unspliced and (optionally) spliced alignments of reads, respectively. We also compare the results against deBGA, an aligner that also utilizes an index built over the compacted de Bruijn graph.

We analyze the performance of these tools on both simulated and experimental DNA and RNA sequencing datasets. The accuracy of PuffAligner is comparable to Bowtie2, which exhibits very high alignment. PuffAligner generally performs better than STAR and deBGA (though, unlike STAR, none of these other tools currently support spliced read alignment). In terms of speed and memory, PuffAligner reaches a tradeoff between the relatively high memory usage of STAR and deBGA and the slower speed of Bowtie2. Hence, while the memory requirement of PuffAligner is more than that of Bowtie2, the speed gain is significant. In the tests performed in this manuscript, PuffAligner is almost always the fastest tool (with the exception being that STAR is faster when aligning unspliced DNA-seq reads to a single human genome).

An additional advantage of the Pufferfish index used in PuffAligner is that it can scale well to a collection of genomes, transcriptomes, or both, especially when the reference sequences being indexed exhibit a high degree of sequence similarity. This feature is already utilized in a specific pipeline for RNA-seq quantification that makes use of a joint index over the genome and transcriptome (Srivastava *et al.*, 2020). The analysis shows that specificity of alignments in such a case can be improved by filtering from quantification reads that are better aligned to some genomic locus that is not present in the transcriptome.

Furthermore, the nature of the Pufferfish index, that explicitly factorizes out highly repetitive sequence, coupled with the fast (and repetition-aware) alignment procedure of PuffAligner makes it a particularly useful for indexing and aligning to a highly similar collection of sequences. This potentially makes it a good match for metagenomic analyses.

We have provided a proof of concept for such a PuffAligner-based metagenomic analysis pipeline, and plan to build a more sophisticated and fully featured metagenomic analysis framework around PuffAligner in the future.

## Supplementary Material

btab408_Supplementary_Data
