# Supplementary material for: PuffAligner: a fast, efficient and accurate aligner based on the Pufferfish index
Source: Bioinformatics. 2021 Jun 12;37(22):4048–55. doi: 10.1093/bioinformatics/btab408 (PMC9502150; doi:10.1093/bioinformatics/btab408)
Supplement: btab408_Supplementary_Data [file btab408_supplementary_data.pdf]

Supplementary Material for:

"Puffaligner : A Fast, Efficient, and Accurate Aligner Based on the Pufferfish Index"

Fatemehalsadat Almodarresi, Mohsen Zakeri and and Rob Patro

1 Enhancing alignment computation

By only aligning the read's sub-sequences that are not included in the MEMs, the size of alignment problems being solved in PuffAligner are often much shorter than the length of the read. However, to further speed up alignment, we also incorporate a number of other techniques to improve the performance of the alignment calculation. We describe the most important of these below:

- **Skipping alignment calculation by recognizing perfect chains and alignment caching:** It is possible to avoid the alignment computation completely in a considerable number of cases. In fact, as has been explained (Sarkar *et al.*, 2018), the alignment calculation step can be completely skipped if the set of exact matches for each chain covers the whole read. PuffAligner skips alignment for cases where the coverage score of chains of MEMs is the length of the read, and assigns a total matched CIGAR string for that alignment. Alignment computation of a read might be also skipped if the same alignment problem has been already detected and computed for this read. For example, in the case of RNA seq data, reads often map to the same exons on different transcripts. In such cases, each alignment solution for a read is stored in a cache (a hash table) so that if the same alignment problem is detected, the solution can be directly retrieved from the cache, and no further computation is required (see supplementary table 1).

Table 1: The percentage of aligner engine calls skipped in the alignment calculation pipeline.

sample	Cache Hits	Perfect Chains	None Alignable	Total Skipped
DNA-seq experimental	52.89%	19.01%	0.71%	72.67%
RNA-seq simulated	28.69%	50.80%	0.97%	80.46%
Metagenomic simulated	61.10%	31.33%	0.00 %	92.43%

- **Early stopping of the alignment computation when a valid score cannot be achieved:** While care is taken to produce only high-scoring chains between the read and reference, it is nonetheless the case that the majority of the chains do not lead to an alignment of acceptable quality. Since the minimum acceptable alignment score is immediately known based on τ and the length of the read, the base-to-base alignment calculation can be terminated at any point where it becomes imposible for the minimum required alignment score to be obtained. This approach can be applied both during the KSW2 alignment calculation, and also after the alignment calculation of each gap is completed. During this procedure, for each base at position i, starting from position 1 on the read of length n, if the best alignment score p up to the i-th position is s_i, we can calculate the maximum possible alignment score, s_{max}, that might be achieved starting at this location given the current alignment score by:

$$s_{max} = s_i + MS * (n - s_i), \tag{1}$$

where MS is the score assigned to each match. If s_{max} is smaller than minimum required score for accepting the alignment, the alignment calculation can be immediately terminated, since it is already known that this anchor is not going to yield a valid alignment for this read.

- **Full-sensitivity banded alignment:** KSW2 is able to perform banded alignment to make alignment calculation more efficient. In this mode, the dynamic programming matrix for the alignment problem is only filled out along the subdiagonals out to a certain distance d away from the main diagonal. If one is guaranteed that any valid alignment must have fewer than d insertions or deletions, then the alignment must not exit these bands of the dynamic programming matrix. Note that alignments with $>d$ indels can be represented within these bands as insertions and deletions move in opposite anti-diagonal directions, but it is certainly the case that no alignment with $\leq d$ indels can exit these bands. By calculating the maximum number of gaps (insertions or deletions) allowed in each sub-alignment probem, in a way that we are certain that any alignment having greater than this number of gaps must drop below the acceptable threshold, we utilize the banded alignment in KSW2 within each sub-alignment problem without losing any sensitivity with respect to non-banded alignment.

2 Orphan recovery

If there is no valid paired-end alignment for a fragment (either concordant or discordant, if the latter is allowed), then PuffAligner will attempt to perform orphan recovery. The term "orphan" refers to one end of paired-end read that is confidently aligned to some genomic position, but for which the other read end is not aligned nearby (and paired). To perform orphan recovery, PuffAligner examines the reference sequence downstream of the mapped read (or upstream if the mapped read is aligned to the reverse complement strand) and directly performs dynamic programming to look for a valid mapping of the unmapped read end. For this purpose, we use the "fitting" alignment functionality of edlib (Šošić and Šikić, 2017) to perform a simple Levenshtein distance based alignment that will subsequently be re-scored by KSW2. Finally, if, after attempting orphan recovery, there is still no valid paired-end mapping for the fragment, then orphan alignments are reported by PuffAligner (unless the "`--noOrphans`" flag is passed).

3 Configurations of aligners in the experiments[

] The performance of each tool is impacted by the different alignment scoring schemes they use, e.g. different penalties for mismatches, and indels. To enable a fair comparison, we attempted to configure the tools so as to minimize divergences that simply result from differences in the scoring schemes. For the experiments in this paper, we use Bowtie2 in a near-default configuration (though ignoring quality values), and attempt to configure the other tools, as best as possible, to operate in a similar manner.

For simplicity and uniformity, all the experiments (except the experiment with simulated DNA-seq reads in the presence of variation) have been run in the concordant mode for both PuffAligner and Bowtie2 (both of which support such an option), disallowing orphans and discordant alignments.

The deBGA scoring scheme is not configurable, so we use this aligner in the default mode (unfortunately, the inability to disable local alignment and forcing just computation of end-to-end alignments in deBGA makes certain comparisons particularly difficult). For PuffAligner we use a scheme as close to Bowtie2 as possible. The maximum possible score for a valid alignment in Bowtie2 is 0 (in end-to-end mode) and each mismatch or gap subtracts from this score. Bowtie2 uses an affine gap penalty scoring scheme, where opening and extending a gap (insertion or deletion) have a cost of 5 and 3 respectively. For DNA-seq reads, we configure STAR to allow as many mismatches as Bowtie2 and PuffAligner by setting the options "`--outFilterMismatchNoverReadLmax 0.12`" and "`--outFilterMismatchNmax 1000`". Also, we use the option "`--alignIntronMax 1`" in STAR to perform non-spliced alignments while aligning genomic reads. For RNA-seq reads, STAR has a set of parameters which we change in our result evaluations, and which are detailed below in the relevant sections.

In Bowtie2 we also use the option `--gbar 1` to allow gaps anywhere on the read except within the first nucleotide (as the other tools have no constraints on where indels may occur). Furthermore, for consistency, we also run Bowtie2 with the option "`--ignore-quals`", since the other tools do not utilize base qualities when computing alignment scores. So, we chose to ignore qualities since that makes it easy to better normalize the scoring functions/ thresholds across methods, but we observe that setting this option has negligible impact on the quality of the alignments found in the simulated samples (see fig. 5).

For quantification of simulated metagenomic samples. all aligners are run in three different configurations, allowing three specific maximum numbers of alignments per fragment; 1 (primary output with highest score, breaking ties randomly), 20, and 200. PuffAligner and STAR, as the only tools that support this option, also are run in the *bestStrata* mode. Further details about configuration of different aligners is explained in supplementary section 3.

concordant-mode: All aligners are configured to report only concordant alignments, i.e., only pairs of alignments that are cocordant and within the "maximum fragment length" shall be reported. The maximum fragment length in all aligners is set to 1000, using the option `--alignMatesGapMax 1000` in STAR, `--maxins 1000` in Bowtie2 and `-u 1000 -f 0` in deBGA. The default value for the maximum fragment length in PuffAligner is set to 1000, the user can cofigure this value by using the flag `--maxFragmentLength`. This concordance requirements also prevents Bowtie2, PuffAligner, and STAR from aligning both ends of a paired end read to the same strand.

Removing highly repetitive hits: As explained in section 2.1, for the sake of performance, highly repeated anchors (more than a user-defined limit) will be

discarded before the alignment phase. This threshold is by default equal to 1000 in PuffAligner. We set the threshold to the same value for STAR and deBGA using options `--outFilterMultimapNmax 1000` and `-n 1000` respectively. There is no such option exposed directly in Bowtie2.

End-to-end alignment: Since PuffAligner finds end-to-end alignments for the reads, we are also running other tools in end-to-end mode, which is the default alignment mode in Bowtie2 as well. In STAR we enable this mode using the option `--alignEndsType EndToEnd`. In the case of deBGA, although the documentation suggests it is *not* supposed to find local alignments by default, the output SAM file contains many reads with relatively long soft clipped ends, so if a read is not aligned end-to-end, deBGA reports the local alignment for that. We were not able to find any option to force deBGA to perform end-to-end alignments for all reads, and so we have compared it in the configuration in which we were able to run it.

Configuring the number of output alignments per read: For aligning DNA-seq samples, each aligner is configured to report a single alignment, which is the primary alignment, for each read. Bowtie2 outputs one alignment per read by default. To replicate this in the other tools, we use the option `--outSAMmultNmax 1` in STAR, `-o 1 -x 1` in deBGA, and `--primaryAlignment` in PuffAligner.

For aligning RNA-seq reads, PuffAligner, by default, outputs up to 200 alignments per read. Bowtie2 is run with the option `-k 200` which lets the tool output up to 200 alignments per read. The value of 200 is adopted from the suggested parameters for running RSEM (Li and Dewey, 2011) with Bowtie2 alignments. We note that running Bowtie2 with this option makes the tool considerably slower than the default mode, as many more alignments will be computed and output to the SAM file under this configuration. deBGA is also run in the with options `-o 200 -x 200`, which nominally has the same effect as `-k 200` in Bowtie2, according to the documentation of deBGA.

Scoring scheme in PuffAligner: PuffAligner, by default, uses a match score of 2 and mismatch penalty of 4. For indels, PuffAligner uses an affine gap scoring schema with gap open penalty of 5 and gap extension penalty of 3. In PuffAligner, after computing the alignment score for each read, only the alignments with a score higher than τ times the maximum possible score for the read are reported. The value of τ is controlled by the option *–minScoreFraction*, which is set to 0.65 by default.

4 Accuracy Measurements

The formula for calculating the metrics used for evaluating the abundance estimation results in the manuscript are as follows. The metrics are Mean Absolute Relative Difference (MARD), Mean Absolute Error (MAE), and Mean Squared Log Error (MSLE). Each metric measures different characteristics of the predicted versus true abundance estimates. For example, lower MARD indicates better distribution of the reads among the references relative to the abundance of each reference, while MAE shows the quality of the distribution of the reads in a more absolute way

regardless of the difference between the abundance of the references. In this case, one misclassified read has the same impact on the MAE metric both for a high-abundance and low-abundance reference.

$$\mathrm{MARD}(y,\hat{y}) = \frac{1}{n_{\mathrm{refs}}} \sum_{i=0}^{n_{\mathrm{refs}}-1} \begin{cases} \frac{|y_i - \hat{y}_i|}{y_i + \hat{y}_i}, & \text{if } y_i + \hat{y}_i > 0 \\ 0, & \text{otherwise} \end{cases}.$$

$$\mathrm{MAE}(y,\hat{y}) = \frac{1}{n_{\mathrm{refs}}} \sum_{i=0}^{n_{\mathrm{refs}}-1} |y_i - \hat{y}_i|. \tag{2}$$

$$\mathrm{MSE}(y,\hat{y}) = \frac{1}{n_{\mathrm{refs}}} \sum_{i=0}^{n_{\mathrm{refs}}-1} (y_i - \hat{y}_i)^2.$$

5 Alignment of DNA-seq reads

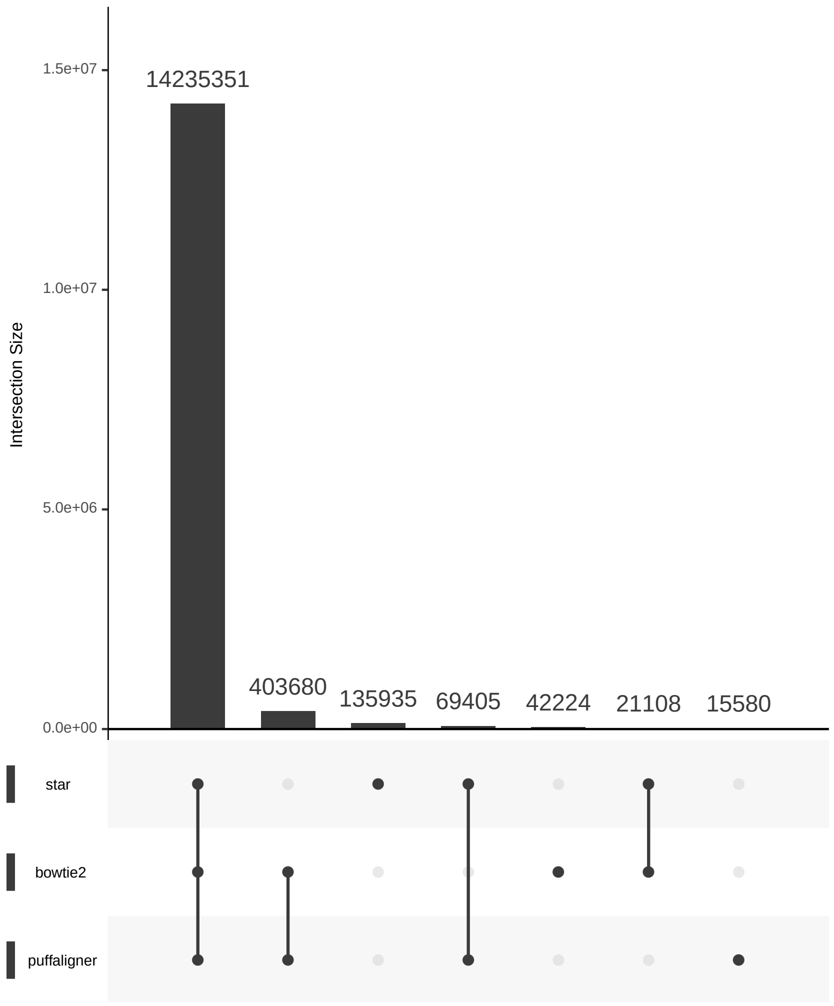

Figure 1: Upset plot showing the agreement of the alignments found by different tools.

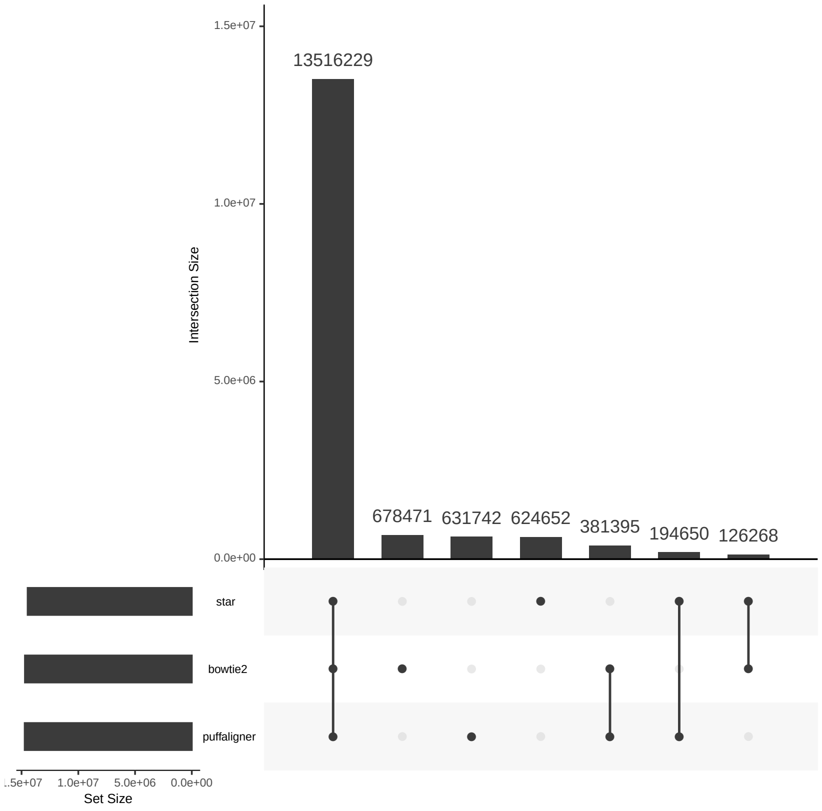

Figure 2: Upset plot showing the agreement of the alignments found by different tools based on the location of the mappings

The value passed to the `--offrate` flag in Bowtie2 governs the fraction of Burrows-Wheeler rows that are sub-sampled to be stored explicitly in the FM-index, the marked (sub-sampled) rows of the BWT might be queried directly during alignment. The BWT value for the other non-marked rows are computed in query time based on the relative position of the unknown rows from the closest marked row. The size of the FM-index depends on the number of BWT rows which are marked. By default in Bowtie2, the “offrate” parameter is set to 5, which marks every $2^5 = 32$ rows of the BWT. Setting a smaller offrate parameter increases the size of the Bowtie2’s index. This enables direct access to more BWT rows which might affect the alignment speed. We evaluated how different offrate values affect the Bowtie2’s index size and alignment speed for aligning a DNA-seq sample (‘ERR013103’) to the human genome. We used offrate values from 0 (no sub-sampling) to 5 (the default value) to build the Bowtie2’s index and align the DNA-seq sample using each index separately. The alignment runtime and maximum memory footprint as well as the index size for different offrate parameters are shown in fig. 3. The plot in this figure suggests that using smaller offrate values increases the Bowtie2’s index size significantly which causes a significant rise in the maximum memory footprint as well. However, smaller offrate values have a small reduction on Bowtie2’s runtime for aligning this DNA-seq sample.

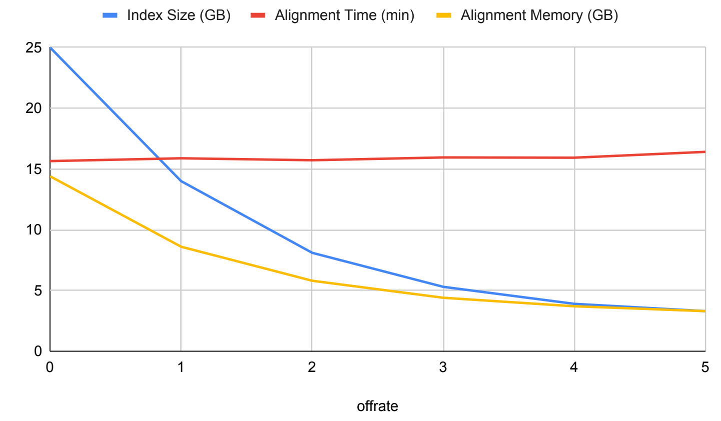

Figure 3: Performance of Bowtie2 with different configurations of the offrate parameter from 0 to 5. Using smaller offrate values increases the size of the index and maximum memory footprint in Bowtie2 while having slight reduction the alignment speed.

6 Transcript abundance estimation from RN-seq reads

We generated 9,968,245 paired-end RNA-seq reads using the polyester (Frazee *et al.*, 2015) read simulator. The reads are generated by the `simulate experiment count-mat` module in polyester. The input count matrix is calculated based on the estimates

from the Bowtie2-Salmon pipeline on the sample `SRR1085674` (where reads are first aligned with Bowtie2 and then the alignments are quantified using Salmon). This sample is a collection of paired-end RNA-seq reads sequenced from human transcriptome using an Illumina HiSeq (Lonsdale *et al.*, 2013). The human transcriptome from gencode release (33) is used to build all the aligners' indices. Also, for building STAR's index in the genome mode, the human genome and the comprehensive gene annotation (main annotation file) is obtained from the same release of gencode.

As the reads in this experiment are RNA-seq reads sequenced from the human transcriptome, it is important to account for multi-mapping, as often, a read might map to multiple transcripts which share the same exon or exon junction. This property makes the direct evaluation of performance at the level of alignments difficult. Therefore, a typical approach in evaluating the accuracy of the transcriptomic alignments is to assess the accuracy of downstream analysis such as abundance estimations by computing the correlation and relative differences of the estimates with the true abundance of the transcripts. To compare the accuracy of each tool we give the alignments produced by each aligner, which are in the SAM format, as input to Salmon to estimate the transcript expressions.

To enable the multi-mapping to take into account the characteristics of alignment to the transcriptome, all tools are configured to output up to 200 alignments per read. Furthermore, the aligners, if possible, are run in the concodrdant-mode in this experiment.

We ran STAR with the `'ENCODE'` options, which are recommended in the STAR manual for RNA-seq reads. STAR is run in two different modes, one is by building the STAR index on human genome, while it is also provided a GTF file for gene annotation. In this mode, STAR performs spliced alignment to the genome, then projects the alignments onto transcriptomic coordinates. The other mode is building the STAR index on the human transcriptome directly, which allows STAR to align the RNA-seq reads directly to the transcripts in an unspliced manner. We chose to run STAR in the transcriptomic mode as well, since we find that it yields higher accuracy, though this increases the running time of STAR.

Accuracy of abundance estimation by Salmon, when provided the SAM output generated by each aligner, is displayed in Table 2. We have used Spearman correlation, and the Mean Absolute Relative Difference (MARD), as explained in Supplementary Equation (2), to evaluate the accuracy of the abundance estimates based on the different alignments. The timing and memory benchmarks provided in this table is only for the alignment step. Alignments produced by PuffAligner, Bowtie2 and STAR in the transcriptomic mode produce the best abundance estimates. deBGA's output alignments are not suitable for any abundance estimation as many reads are aligned only to the same strand which are later filtered during the abundance estimation by Salmon, so we could not provide a meaningful correlations for abundance estimation using deBGA's alignments. Aligning the reads by STAR to genome and then projecting to transcriptomic coordinates does not generate as high correlation as directly aligning the reads to the transcriptome by STAR. However, we note that, as described by Srivastava *et al.* (2020), there are numerous reasons to consider alignment to the entire genome that are not necessarily reflected in simulated experiments. While the memory usage by PuffAligner is only 2 fold larger

than memory used by Bowtie2, it computes the alignments much more quickly.

According to the results in table 2 PuffAligner is the fastest aligner in these benchmarks, and the accuracy as high as Bowtie2 and STAR for aligning RNA-seq reads. Here, PuffAligner leads to the most accurate abundance estimates, while being 25 times faster than Bowtie2. Moreover, The memory usage is much less than other fast aligners such as STAR.

aligner	spearman	MARD	time(m:s)	memory(GB)
PuffAligner	0.92	0.05	1:17	2.54
deBGA	N/A	N/A	5:19	9.96
STAR- txp	0.92	0.05	1:57	8.73
STAR	0.90	0.06	3:30	32.57
Bowtie2	0.92	0.05	32:59	1.15

Table 2: Abundance estimation of simulated RNA-seq reads, computed by Salmon, using different tools' alignment outputs. The time and memory are only for the alignment step of each tool and the time for abundance estimation by Salmon is not considered.

7 Alignment of simulated DNA-seq reads in the presence of variation

To further investigate the accuracy of the aligners, we used simulated DNA-seq reads. One of the main differences between simulated reads and experimental reads is that simulated reads are often generated from the same reference sequences to which they are aligned, with the only differences being due to (simulated) sequencing error. While (simulated) sequencing error prevents most reads from being exact substrings of the reference, it actually does not tend to complicate alignment too much. On the other hand, while dealing with experimental data, the genome of the individual from which the sample is sequenced might include different types of variations with respect to the reference genome to which we are aligning (Srivastava *et al.*, 2020). Therefore, it is desirable to introduce variations in the simulated samples, and to measure the robustness and performance of the different aligners in the presence of the variation. Mason (Holtgrewe, 2010) is able to introduce different kinds of variations to the reference genome, such as SNVs, small gaps, and also structural variants (SV) such as large indels, inversions, translocations and duplications. We use Mason to simulate 9 DNA-seq samples with different variation rates ranging from $1e{-}7$ to $1e{-}3$. Each sample includes $1M$ paired-end Illumina reads of 100bp length from chromosome 21 of the human genome, ensembl release 98[1].

[1] ftp://ftp.ensembl.org/pub/release-98/fasta/homo_sapiens/dna/

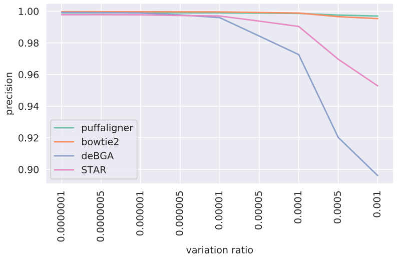

(a) The precision of the alignments reported by each aligner. True positives (TP) are the compatible reads that are aligned to the original location, and the FP set consists of both the compatible reads aligned to sub-optimal locations with larger edit distance than the alignment to the original location and the non-compatible reads that are aligned with high (>25) edit distance.

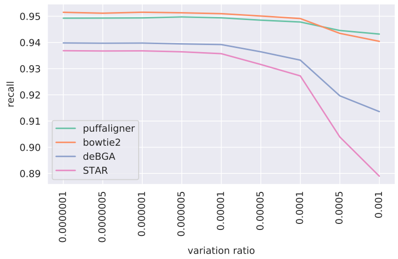

(b) The ratio of the alignments in the true SAM file that are recovered by each aligner. The recall is the result of dividing the number of TP reads by the total number of compatible reads.

Figure 4: Comparing the accuracy of aligners in the presence of different rates of variations in the reference genome

For this analysis, we do not restrict the aligners to only report concordant alignments, since the structural variations in the samples can lead to valid discordant alignments, such as those on the same strand or with inter-mate distances larger than the maximum fragment length. To be specific, we do not use the options which limit Bowtie2 and PuffAligner to report only concordant alignments, in addition, we use the option "`--dovetail`" in Bowtie2 to consider dovetail pairs as concordant pairs.

The alignments reported by deBGA already include discordant pairs and also orphan mappings. Furthermore, To remove any restrictions on the fragment length in the alignments reported by deBGA, we set the minimum and maximum insert size, respectively to 0 and the 50000, since setting a larger value resulted in the tool running into segmentation fault.

To allow dovetail pairs and also larger gaps between the pairs in STAR, we use the following options: "`--alignEndsProtrude 1000000 ConcordantPair`", "`--alignMatesGapMax 1000000`". By default there is not a specific option in STAR for allowing orphan alignment of paired end reads. Instead, we can increase the number of allowed mismatches to be as large as one end of the read by using the following options: "`--outFilterMismatchNoverReadLmax 0.5`", "`--outFilterMismatchNoverLmax 0.99`", "`--outFilterScoreMinOverLread 0`", "`--outFilterMatchNminOverLread 0`".

For each sample, Mason produces a SAM file which includes the alignment of the simulated reads to the original, non-variant version of the reference — the version which was used for building the aligner's indices in this experiment. Based on the alignments reported in the truth file, some reads did not have a valid alignment to the original reference. This was the result of a high rate of variations at some

sequencing sites. We called the set of reads that, according to the truth SAM file, were aligned to the original reference as compatible reads.

We compared the performance of aligners based upon how well they are able to align the compatible reads. Note that we evaluate the alignment for each end of the reads independently. We computed the precision and recall of the alignments reported for these reads as follows. True positives are considered the reads that are mapped by the aligner to the same location stated by the truth file. Then, recall is computed by dividing the number of true positives by the number of all compatible reads. Furthermore, we considered an alignment as a false positive in two different cases. First, for the non-compatible reads if the aligners report an alignment with edit distance larger than 25, it will be considered a false positive. Second, in the case that an aligner reported an alignment to a location other than the one in the truth file, it was considered as a false positive if the edit distance of the reported alignment is greater than the edit distance of the true alignment. Having defined the set of TP and FP for the alignments, and also having considered the set of all compatible reads as the set we are trying to recover, we computed precision and recall for the set of alignments reported by each aligner.

Figure 4 shows the precision and recall of the aligners for different samples. According to fig. 4, for lower variation ratios up until $10e-5$, most of the tools are able to make accurate alignment calls with a high specificity. As the variation ratio introduced in the sample is increased, all the tools start to have lower precision and recall. deBGA and STAR perform worse in higher variation samples, as they fail to recover the true alignment for more reads, while Bowtie2 and PuffAligner are able to align most of the reads to their true location on the original reference.

These results show that PuffAligner' accuracy is stable in the face of variation which makes the tool suitable for datasets that are known to have substantial variation, such as when aligning reads to microbial genomes where the specific sequenced strain may not be represented in the reference set.

To test whether enabling the quality awareness affects the accuracy of the alignments found by Bowtie2 in these simulated samples, we have run Bowtie2 in both (with and without "`--ignore-quals`" flag) and the results are provided in fig. 5. These plots suggest that setting this option has negligible impact on the quality of the alignments found by Bowtie2 in the simulated samples.

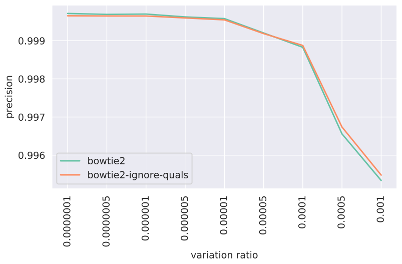

(a) The precision of the alignments reported by Bowtie2 whith and without quality awareness. True positives (TP) are the compatible reads that are aligned to the original location, and the FP set consists of both the compatible reads aligned to sub-optimal locations with larger edit distance than the alignment to the original location and the non-compatible reads that are aligned with high (>25) edit distance.

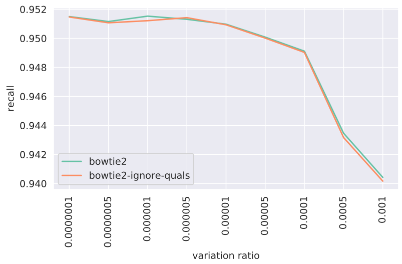

(b) The ratio of the alignments in the true SAM file that are recovered by Bowtie2 with and without quality awareness. The recall is the result of dividing the number of TP reads by the total number of compatible reads.

Figure 5: Comparing the accuracy of alignments found by Bowtie2 with and without quality awareness

8 Alignment of single-strain Simulated reads to an Index of Highly Similar Strains

For this experiment, we download the viral database from NCBI, and choose three similar coronavirus genomes. This set includes one of the recently-uploaded samples from Wuhan (Wu *et al.*, 2020; Baranov *et al.*, 2005). We select three very similar viral genomes to simulate reads from, which are: NC_045512.2, NC_004718.3, and NC_014470.1. There are also a lot of literature discussing the similarity in sequence and behavior for these three species of coronavirus (Wang *et al.*, 2020; Zhang *et al.*, 2020; Tang *et al.*, 2020). The first is the complete genome for severe acute respiratory syndrome coronavirus 2 isolate Wuhan-Hu-1 known as Covid19 with length of 29,904 bases. NC_004718.3 is the ID of SARS coronavirus complete genome (length: 29,752) and finally, NC_014470.1 is a Bat coronavirus BM48-31/BGR/2008 complete genome (length: 29,277).

We use Mason (Holtgrewe, 2010) to generate three simulated samples, each sample contains 500,000 reads only from one of the three viral references we mentioned earlier. Then, reads were aligned back to the database of viral sequences using each of the four aligners. The results are shown in table 3. As the results show, the alignments of all aligners, except for deBGA, are distributed only across the three references of interest out of all the reference sequences in the complete viral database. deBGA reports only a few alignments to a forth virus. In general, all of the aligners do a good

job of reporting the correct alignment among the returned alignments for each read.

Here, we are more interested in exploring how sub-optimal alignments are computed and filtered under different settings when aligning to a collection of very similar genomes. The results show that all tools have very high sensitivity even when considering only a single (primary) alignment per read. As we allow more alignments to be reported, the sensitivity increases and quickly levels off for all the tools. On the other hand, more alignments are generated and Bowtie2, in particular, generates a considerable number of extra alignments as the maximum number of allowed reported alignments is increased. However, the results do not change when allowing more than 20 alignments, which means no more than 20 alignments ever pass the alignment score threshold for these reads in the viral database for any of the tools we are testing.

The results indicate that, when allowing more than one alignment to be reported for every read, Bowtie2 tends to report a large number of sub-optimal (yet, still valid) alignments compared to other tools. These are alignments that are accepted within the alignment score threshold, but are to another target than the one from which the read truly originates. Generating these sub-optimal alignments is in no way wrong, but it has a non-trivial computational cost, as shown in figure 6, even if these alignments are not used in downstream analysis. Further, the score of the best alignment for each read is specific to that read and not known ahead of time, meaning that this situation cannot be completely addressed simply by setting more stringent parameters for which alignment scores should be allowed. This behavior of Bowtie2 gives the other tools a computational advantage when the user only truly requires the set of equally-best alignments for each read.

Interestingly, there is one read that all tools, except for PuffAligner fail to properly align. Inspecting this alignment reveals it is a valid alignment within the range of the acceptable scoring threshold, and it is unclear why it is not discovered by the other tools. Overall, the aligners tested perform very well here in reporting the true strain of origin without reporting too many extra alignments. Interestingly, despite changing the parameters to allow more alignments, STAR tends to return the same set of alignments under all configurations in this experiment. Figure 6 compares the running time of different tools for aligning the single strain microbiome samples.

BestStrata Mode: In this small example, all tools showed good sensitivity (and PuffAligner and STAR showed near-perfect sensitivity) even when reporting only a single-alignment per read. This experiment is, of course, an atypically small test for multi-mapping read. In in larger samples, with reads deriving from more organisms and a larger database of references, permitting more alignments usually yields non-trivial improvements in sensitivity. To control the rate of reporting sub-optimal alignments, PuffAligner supports the "best strata" option – also available to STAR, which allows only the alignments with the best calculated score to be reported (as a replacement for maximum allowed number of alignments). Using this option, PuffAligner achieves full specificity and sensitivity in this experiment 3. The same results are achieved for the other two simulated single-strain samples shown in the supplementary table 3. We further demonstrate the positive impact of this option on the alignment of bulk metagenomic samples in the next section.

Table 3: Alignment Distribution for three samples of 500,000 simulated reads from reference sequences NC_045512.2 (known as covid19), NC_004718.3 (known as SARS Coronavirus) and NC_014470.1 (Bat Coronavirus assembled in 2008) respectively. The best specificity is achieved by PuffAligner in `bestStrata` mode (as well as the primary mode). In these simulated sample, many alignments are not ambiguous, resulting in the good performance observed when using only primary alignments. However, typically in metagenomic analysis, many equally-good alignments exist, and selecting only one is equivalent to making a random choice.

Sample's Origin	Alignment Mode	Tool	NC_045512.2 (Covid19)	NC_004718.3 (SARS Coronavirus)	NC_014470.1 (Bat Coronavirus)	Other Assembled References
COVID19	Primary	PuffAligner	**500,000**	**0**	**0**	**0**
		Bowtie2	499,981	18	**0**	**0**
		STAR	499,999	**0**	**0**	**0**
		deBGA	499,991	**0**	**0**	9
	Up to 20	PuffAligner	**500000**	134	46	**0**
		Bowtie2	499,999	21,461	2,311	**0**
		STAR	499,999	**0**	**0**	**0**
		deBGA	499,991	**0**	**0**	9
	Up to 200	PuffAligner	**500000**	134	46	**0**
		Bowtie2	499,999	21,461	2,311	**0**
		STAR	499,999	**0**	**0**	**0**
		deBGA	499,991	**0**	**0**	9
	Best strata	PuffAligner	**500000**	**0**	**0**	**0**
		STAR	499,999	**0**	**0**	**0**
SARS	Primary	PuffAligner	**0**	**500,000**	**0**	**0**
		Bowtie2	24	499,975	**0**	**0**
		STAR	**0**	499,998	**0**	**0**
		deBGA	**0**	499,995	**0**	5
	Up to 20	PuffAligner	116	**500000**	486	**0**
		Bowtie2	21,546	499,999	7,205	**0**
		STAR	**0**	499,998	**0**	**0**
		deBGA	**0**	499,995	**0**	5
	Up to 200	PuffAligner	116	**500000**	486	**0**
		Bowtie2	21,546	499,999	7,205	**0**
		STAR	**0**	499,998	**0**	**0**
		deBGA	**0**	499,995	**0**	5
	Best strata	PuffAligner	**0**	**500000**	**0**	**0**
		STAR	**0**	499,998	**0**	**0**
Bat2008	Primary	PuffAligner	**0**	**0**	**500000**	**0**
		Bowtie2	**0**	**0**	499,999	**0**
		STAR	**0**	0	499,999	**0**
		deBGA	**0**	**0**	499,991	9
	Up to 20	PuffAligner	32	494	**500000**	**0**
		Bowtie2	2,343	7,127	499,999	**0**
		STAR	**0**	**0**	499,999	**0**
		deBGA	**0**	**0**	499,991	**0**
	Up to 200	PuffAligner	32	494	**500000**	**0**
		Bowtie2	2,343	7,127	499,999	**0**
		STAR	**0**	**0**	499,999	**0**
		deBGA	**0**	**0**	499,991	9
	Best strata	PuffAligner	**0**	**0**	**500000**	**0**
		STAR	**0**	**0**	499,999	**0**

9 Alignment of Bulk Simulated Metagenomic Experiments

9.1 Simulating Bulk RNA samples

For simulating a bulk metagenomic sample, we generate a list of simulated whole genome sequencing (WGS) reads through the following steps:

- Select a real metagenomic WGS read sample
- Align the reads of the chosen real experiment to the 4000 genomes using Bowtie2, limiting Bowtie2 to output one alignment per read.
- Choose all the references with count greater than C from the quantification results. This defines the read distribution profile that we will use to simulate data.
- For each of the expressed references, use Mason (Holtgrewe, 2010), a whole genome sequence simulator, to simulate $100bp$ paired-end reads with counts proportional to the reported abundance estimates so that total number of reads is greater than a specified value n. In this step we ran Mason with default options.
- Mix and shuffle all of the simulated reads from each reference into one sample which is used as the mock metagenomic sample.

We select three metagenomic experiments to simulate read from; A soil experiment with accession ID `SRR10948222` (Fisher, 2020) from a project for finding sub-biocrust soil microbial communities in the Mojave Desert. The sample has $\sim 27M$ paired-end reads, containing a mixture of genomes from various genera and families. However, less than $200k$ of the reads in the sample were aligned to the strains present in our database, leading the selection of 98 species from a variety of genera. We scaled the read counts in the simulation to $\sim 50M$ reads. The other two selected samples are `SRR11283975` and `SRR11496426` the details of which are explained in 4.

Table 4: Basic information for samples selected for simulating mock bulk metagenomic samples.

Accession	Project description	# of reads	# of reads aligned to 4k selected reference	# of simulated reads	# of references of origin for the simulated reads
SRR10948222	Finding sub-biocrust soil microbial communities in Mojave Desert, California, United States	27,296,270	200k	5,550,650	98
SRR11283975	The impact of different acidification degrees on the bacterial community in the Jiaodong Peninsula, China	35.5k	8,333	1,012,176	92
SRR11496426	The composition, genetics characteristics and structure of the microbial communities of the oil site of Uzon Caldera	42.3k	30,203	1,029,382	179

Table 5: Alignment accuracy of the tools over different accuracy metrics for the mock sample simulated from real samples with accession IDs SRR11283975 and SRR11496426.

Accession ID	Alignment Mode	Tool	Spearman	MARD	MAE	MSLE
SRR11283975 Jiandong Peninsula	Primary	PuffAligner	0.71	0.024	0.43	0.044
		Bowtie2	0.615	0.04	0.64	0.071
		STAR	**0.727**	**0.02**	**0.406**	**0.039**
		deBGA	0.274	0.521	106.78	3.788
	Up to 20	PuffAligner	0.942	**0.003**	0.074	**0.002**
		Bowtie2	0.909	0.005	**0.049**	0.004
		STAR	**0.946**	**0.003**	0.087	**0.002**
		deBGA	0.277	0.489	101.39	3.366
	Up to 200	PuffAligner	**0.979**	**0.001**	**0.068**	0
		Bowtie2	0.97	0.002	0.039	0.001
		STAR	0.951	0.003	0.086	0.001
		deBGA	0.278	0.483	100.961	3.293
	Best strata	PuffAligner	**0.979**	**0.001**	**0.063**	0
		STAR	0.951	0.003	0.086	0.001
SRR11496426 Uzon Caldera	Primary	PuffAligner	**0.568**	**0.112**	32.552	0.953
		Bowtie2	0.53	0.14	38.062	1.101
		STAR	0.559	0.118	**31.823**	**0.825**
		deBGA	0.367	0.566	115.882	3.569
	Up to 20	PuffAligner	**0.78**	**0.03**	7.43	0.239
		Bowtie2	0.74	0.042	10.83	0.304
		STAR	0.713	0.049	**6.939**	**0.165**
		deBGA	0.368	0.554	109.29	3.317
	Up to 200	PuffAligner	0.865	0.017	**5.635**	**0.105**
		Bowtie2	**0.879**	**0.015**	7.208	0.134
		STAR	0.724	0.045	6.496	0.133
		deBGA	0.369	0.549	108.986	3.273
	Best strata	PuffAligner	**0.85**	**0.019**	**5.571**	**0.092**
		STAR	0.723	0.046	6.544	0.134

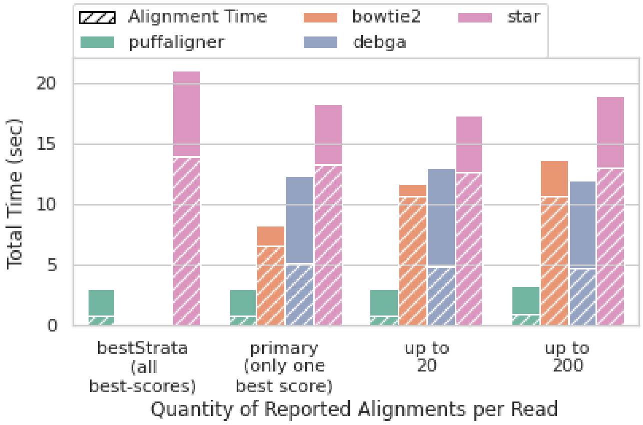

Figure 6: Time performance for aligning a single strain sample averaged over all three alignment processes for the samples covid19, sars, and bat200. The dashed area shows fraction of the time spent purely on aligning reads where the remaining portion is the time required for index loading. PuffAligner is the fastest tool in this experiment, yet most of its time is still dedicated to loading the index.

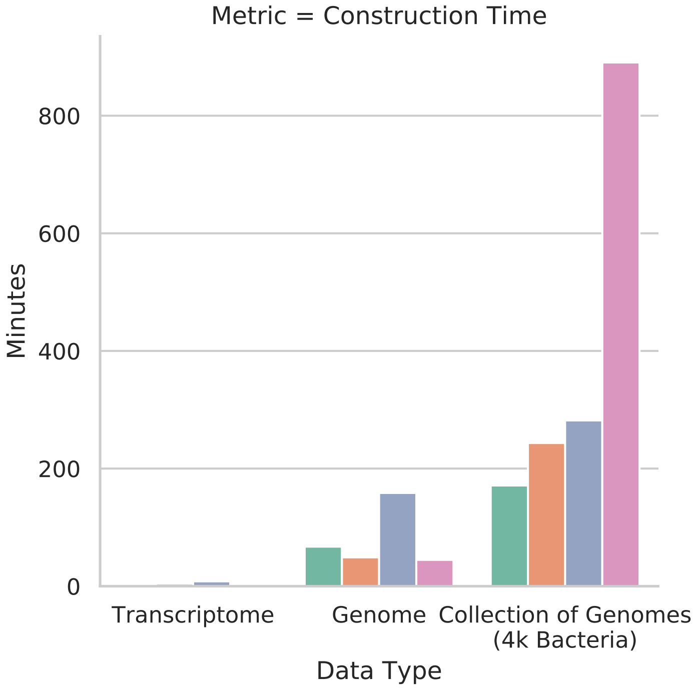

Figure 7: Scalability of different tools over index construction running time for three different datasets, human transcriptome (gencode version 33), human genome (GRCh38 primary assembly), and collection of genomes (4000 random bacterial complete genomes). All tools are run with 16 threads.

10 PuffAligner scalability in the presence of high redundancy

In this experiment, we select two sets of 100 microbial complete genomes from the NCBI bacterial database. In the first set, selected genomes are from distinct bacterial species (which we refer to as the "different genomes" set) and in the second set (which we refer to as "similar genomes" set), genomes are all different strains all belonging to the *Escherichia coli* species with taxa ID 562. The list of bacteria genome accession IDs used for this experiment have been published to Zenodo with DOI "10.5281/zenodo.4569270". We benchmark index construction performance and index size for constructing Bowtie2 and PuffAligner indices over 10, 50, and 100 strains in each of the two sets. Figure fig. 8 shows the Bowtie2 and PuffAligner results in all cases. In all three metrics of index size, construction time and memory, there is no benefit for Bowtie2 related to the similarity of the sequences being indexed. This fully accords with theory, as Bowtie2 makes use of a sampled, but not compressed FM-index. In fact, in this specific experiment, switching from the set of "different species" to set of *E. coli* strains has a negative impact on Bowtie2's index size. On the other hand, PuffAligner performs better on the *E. coli* references (labeled as "similar genomes" in the plots) compared to set of 100 different species, and it shows a better scalability factor (slower growth as the number of references increases) compared to Bowtie2 on *E. coli* references.

(a) Index Size

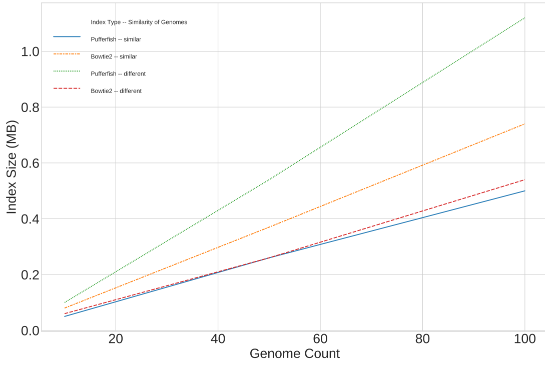

(b) Construction Memory

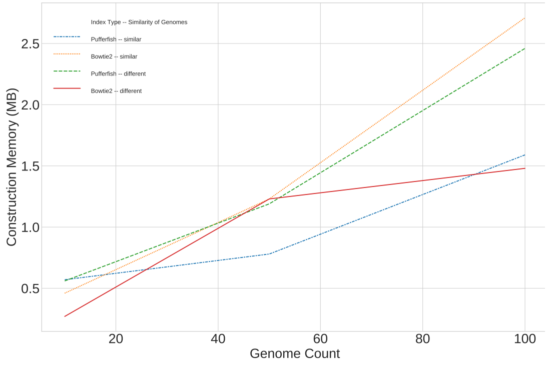

(c) Construction Time

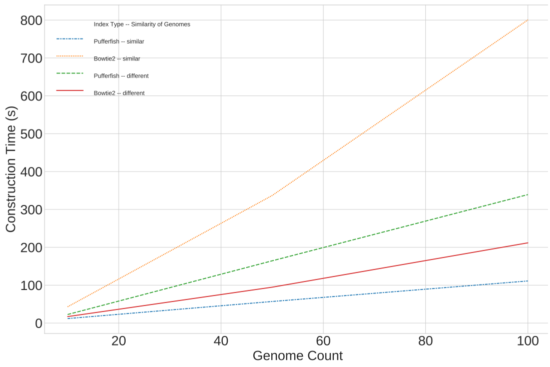

Figure 8: Scalability of PuffAligner and Bowtie2 w.r.t index size, construction memory, and time, when the references in the dataset are highly similar (similar genomes) versus when there is not a high sequence redundancy among the indexed sequences (different genomes).

11 Comparing alignment to a light-weight pipeline like Kraken2 + Bracken

Here, we compare a pipeline for metagenomic abundance estimation comprised of aligning reads using PuffAligner followed by quantification with Salmon, to a k-mer -based abundance estimation method that consists of classifying reads with Kraken2 and estimating the abundances with Bracken. To perform this comparison, we construct an index over all bacteria, viral, archaea, and fungi obtained from

NCBI taxonomy database Federhen (2012) on May 21, 2020 using both Pufferfish and Kraken2. We ran both approaches over 34 samples from different categories of metagenomic analyses. Ten of the samples are selected from non-human projects such as the "metasub project" Consortium *et al.* (2016) as well as from submarine or soil samples Ma *et al.* (2014). The remaining samples are selected from the human metagenome project (hmp) Gevers *et al.* (2012). The human samples are chosen from different tissue categories of plasma, tongue dorsal, gingiva, vaginal, and fecal. We then compare the abundance of the reported references under each of the two pipelines.

We run PuffAligner in two modes; the default mode, which only reports the best concordant alignments, and a less restrictive mode which will be explained. The default mode does not allow any orphan, discordant, or dovetail alignments, filters any mapping with alignment score less than 0.65 fraction of the maximum possible alignment score, i.e., when the read matches the reference perfectly, and for each read reports only alignments with the highest score (bestStrata). We also ran PuffAligner allowing orphans, discordant, and dovetail alignments, but keeping the rest of the parameters as default. We use Salmon to estimate the abundance of references from the alignments. While this is a reasonable pipeline for metagenomic abundance estimation, it is likely that the accuracy could be further improved by incorporating specific features of the metagenomic data in the abundance estimation step, such as the topology of the taxonomy tree and the expression of certain marker genes.

We also run Kraken2 in two different modes. First, we run it in the "default" mode, which allows all reads having any k-mer match to be classified. Second, we run it setting the confidence option to a value c, which prevents reporting reads that have a confidence lower than c. As per the authors' definition[2], this number is calculated based on the ratio of unique k-mers mapped to a taxa in the taxonomy tree over all the non-ambiguous k-mers of the read (k-mers not containing an `N` character). There is not a one-to-one correspondence between the confidence threshold here and the "minScoreFraction" in PuffAligner. However, we believe both of these options are necessary for providing more reliable abundance estimates, by removing the reads that exhibit poor evidence of deriving from an indexed references. Filtering orphaned, discordant, or dovetail reads is a feature only available to the alignment-based procedures and not k-mer counting approaches, since they do not jointly consider such properties of the reads containing the k-mers . That is why we provide both modes for PuffAligner, allowing and disallowing these sort of reads, to provide a fair comparison of the two methods.

In the absence of technical errors and variation from the reference genomes, if a sequencing read comes from a subset of references in the index, then there will be at least one exact match for it. However, due to the presence of both sequencing errors and biological variation of the organisms in the samples with respect to the reference genomes, a perfect alignment does not exist for most reads. Therefore, alignment-based methods try to find the sub-sequences in the reference which matches the read with a high alignment score (including both mismatches and gaps) and report these loci as the potential origin of the read. On the other hand, k-mer -based approaches break the read into its constituent k-mers and treat exact k-mer matches as evidence

[2]https://github.com/DerrickWood/kraken2/wiki/Manual

of a read originating from a reference. In most cases, there is a large degree of concordance between this approach and the alignment-based approaches for finding potential loci (i.e. the number of k-mer matches correlates well with alignment score). Thus, in general, such approaches result in highly-correlated abundance estimates with those produced by alignment-based approaches, and they also tend to be very fast and memory efficient. Nonetheless, such approaches still face challenges as the degree of sequence-similarity between strains (and species) can be very large and, simultaneously, the organisms present in a sample can show considerable sequence divergence from the strains in the reference database. As a result, k-mer -based approaches, while very sensitive, tend to sacrifices specificity in this type of data. In most cases, when no filter is applied to the taxonomic assignments, a k-mer -based approach overestimates the number of reads deriving from the reference strains in each sample.

To see how the reference abundance estimations compare across the two pipelines of Kraken2 and PuffAligner we look at the top 5 most highly-abundant (predicted) species for each sample and their abundance in supplementary fig. 10. We run Kraken2 both with no confidence filter and filtering with a confidence value of .65, and compare the results with default PuffAligner (with minScoreFraction=.65). In certain samples, observe similar highly-abundant species discovered by Kraken2 (with no confidence filter) and PuffAligner that are reported as less abundant when processed with Kraken2 using a confidence threshold of 0.65 (e.g. Streptococcus gordonii for one of the subway samples). However, for other samples, applying the confidence threshold to the Kraken2 results brings the predictions closer to those of PuffAligner (e.g. Lactobocillus Crispatus for two vaginal sample). We do the same analyses at the genus level in fig. 11 and there we observe similar inconsistencies.

To further investigate how the filtering of low quality reads in the two approaches compare, we look at the trend of the number of reads filtered in Kraken2 while applying different confidence thresholds with respect to the default PuffAligner in fig. 9. We run Kraken2 with confidence values of .5, .65, .8, as well as .05 which is recommended by the authors for filtering low quality alignments for general purposes[3]. We compare the results of running the Kraken2 + Bracken pipeline with PuffAligner + Salmon pipeline under both modes of PuffAligner (default and less restrictive) so that the effect of the read-pair-based filters (those taking into account read orphan and dovetail status) can be evaluated. The plots in the top row of fig. 9 show the read count difference of default PuffAligner and Kraken2 applying different confidence values. The total read count varies widely across samples. Therefore, to have a better understanding of the results, we normalize the read count differences based on the PuffAligner reported read count in the two bottom plots of fig. 9.

As is observed, applying the confidence threshold may not cause a uniformly similar impact on the absolute read count for all samples. We expect to see a similar relative effect when applying the threshold over all the samples, i.e. the read count consistently becoming closer to or further from that obtained by the alignment-based approach. Although this is the observed trend for small confidence values which are less effective, the plots in fig. 9 do not show a consistent pattern for larger confidence values in all the samples, where the application of the threshold

[3]https://github.com/DerrickWood/kraken2/issues/167

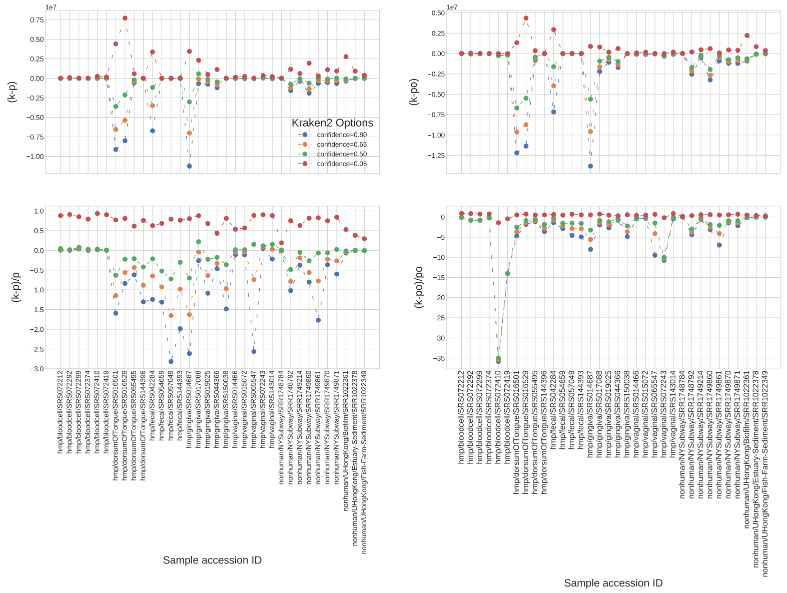

Figure 9: This plot shows the effect of applying different confidence values on the results of Kraken2 + Bracken pipeline relative to PuffAligner in two modes; default, and less restrictive mode (allowing orphan, discordant, or dovetail alignments). The abbreviations on the y-axis labels are defined as follows: k=Kraken2, p=PuffAligner (default), po=PuffAligner (allowing discordant alignments). Applying different confidence values to the Kraken2 pipeline results in an inconsistent pattern of assigned reads across samples when comparing to the PuffAligner read count (for both modes of PuffAligner (default on left and restrictive on right)). The count of filtered reads for both PuffAligner and Kraken2 depends on the sample and quality of the reads (the two plots on the top) as well as the filtered read counts relative to PuffAligner's (the bottom plots). However, there is an observed inconsistency in how changing the confidence threshold of Kraken2 affects the number of filtered reads when comparing to the alignment-based approach of PuffAligner. In fact, not only is there no fixed confidence value that corresponds to the analogous alignment options, but further there is no corresponding value that provides a smooth consistent effect on the number of filtered reads. There are large changes from allocating many more read to allocating many fewer reads compared to PuffAligner in some samples, whereas the allocated read count barely changes in others.

in Kraken2 makes the reported read counts sometimes closer and sometimes farther from the alignment-based pipeline. As might be expected, the read counts reported in Kraken2 are further from the PuffAligner pipeline in the default mode versus when allowing orphans and discordant alignments in the less restrictive mode (as can be seen when comparing the two columns of fig. 9).

To summarize, through the experiments in this section, we observe that the

k-mer -based approaches sometimes result in different reported highly-abundant references than alignment-based approach and, crucially, applying a score filter to the k-mer -based approaches does not always make the abundance predictions for all species closer to the alignment-based predictions. The efficacy of the score filter in the k-mer -based approaches may depend on the technical and biological details particular to that sample, so that the score filter that best matches the alignment-based approach in one sample may not be the same score filter that best matches the alignment-based approach in other samples. This is, perhaps, expected, as the alignment-based approach is taking into account more information, both within and across the ends of a paired-end fragment, than simply the number of matching k-mers .

In this sense, we believe that a sensitive and efficient aligner like PuffAligner, paired with a capable abundance estimation tool, provides a pipeline for metagenomic abundance estimation that, while sacrificing some speed, is likely to be more accurate and robust than k-mer -based approaches.

(a) Bracken with no confidence

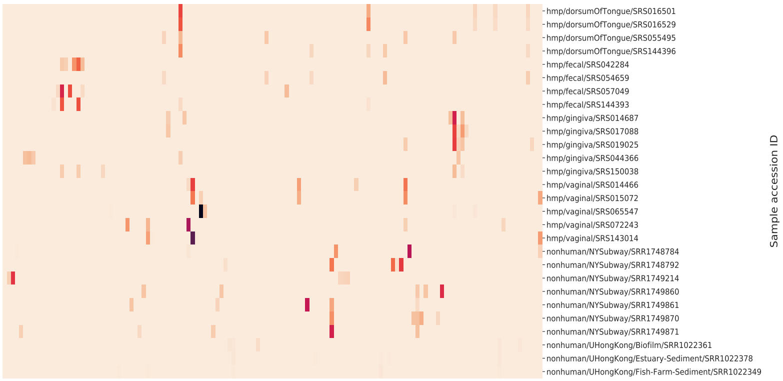

(b) Bracken with con- fi- dence=0.6

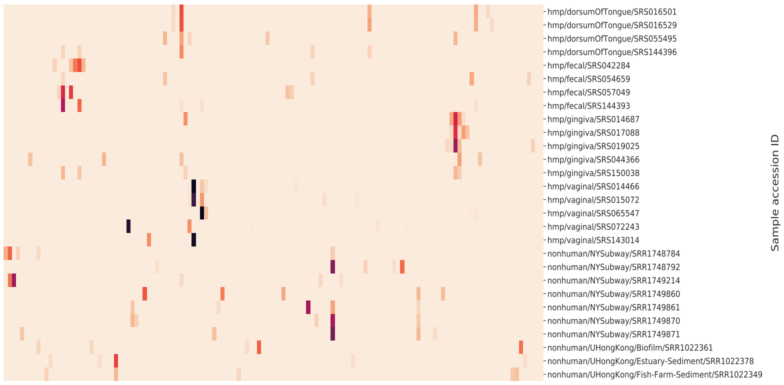

(c) Puffaligne default + Salmon

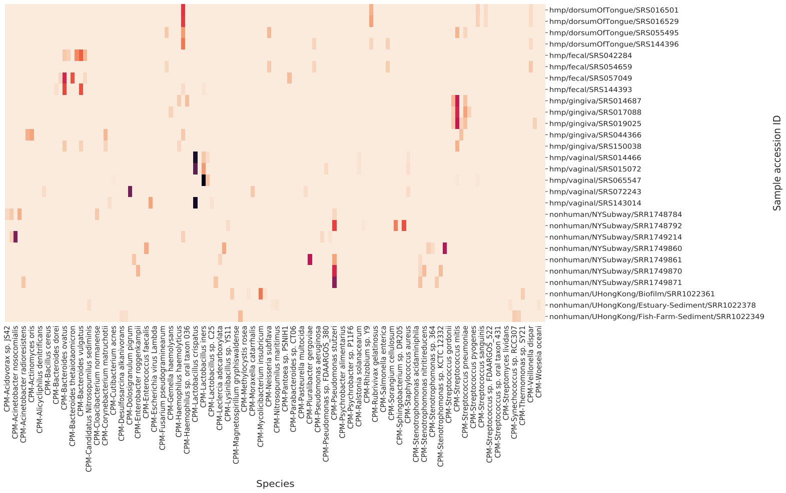

Figure 10: Heatmap showing 5 most popular species over 28 samples through three pipelines of Kraken2(no confidence)+Bracken, Kraken2(confidence=0.65)+Bracken and default Puffaligner+Salmon. Overall, we observe more similarity between Puffaligner and Bracken with confidence of 0.65. However, there are cases where applying the confidence filter to Kraken make the results diverge from Puffaligner pipeline for example "Screptococcus gorbanii" considered abundant in a subway sample in the first and third heatmap, whereas Kraken2(confidence=0.65) does not detect the microorganism as abundant.

(a) Bracken with no confidence

(b) Bracken with confidence=0.6

(c) Puffaligner default + Salmon

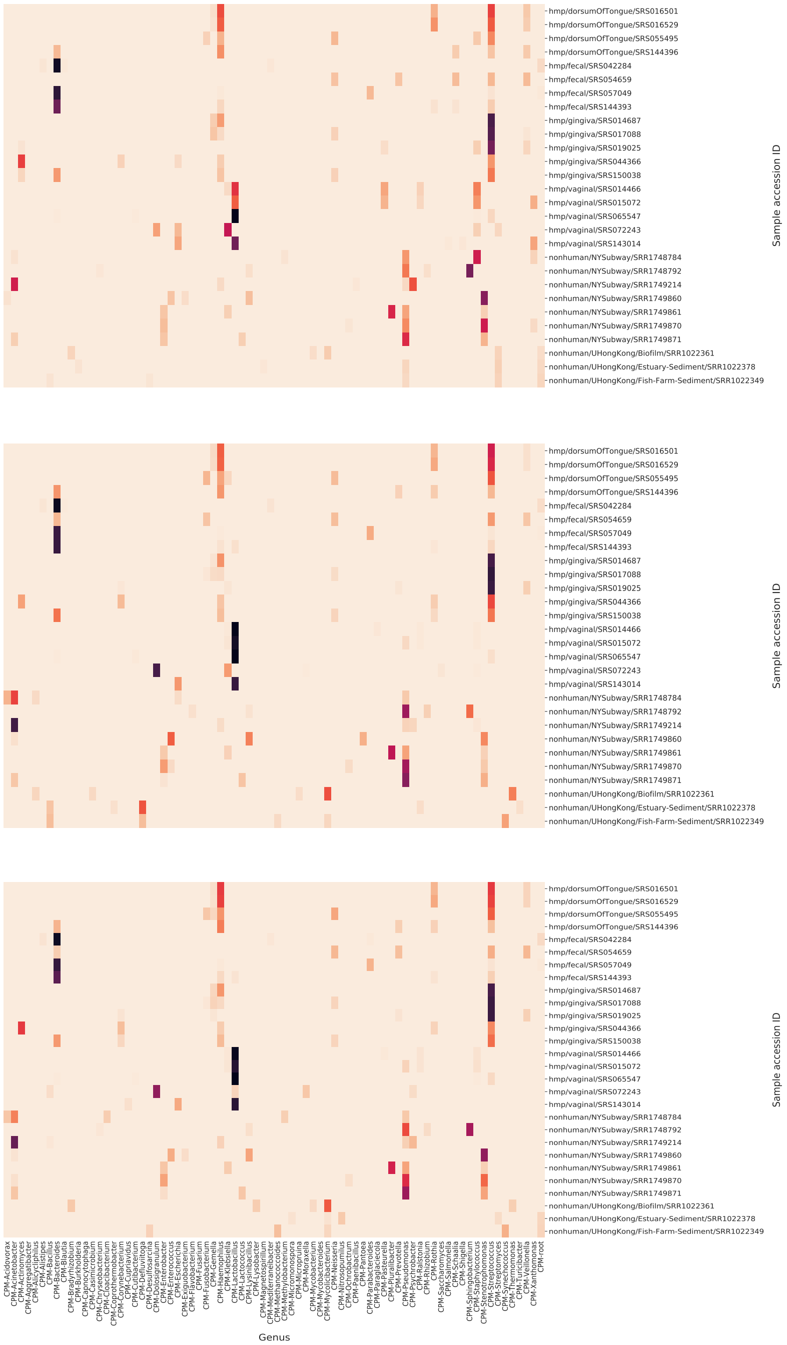

Figure 11: Heatmap showing 5 most popular genera over 28 samples through three pipelines of Kraken2(no confidence)+Bracken, Kraken2(confidence=0.65)+Bracken and default Puffaligner+Salmon.

Table 6: The construction benchmark and final index size for each of the tools over 4000 selected bacterial genomes.

Tool	Time (hh:mm)	Memory (GB)	Index Size (GB)
PuffAligner	**01:40**	61.30	46
deBGA	04:42	129.40	59
Bowtie2	04:03	**50.7**	**27**
STAR	14:50	147.70	134

References

Baranov, P. V., Henderson, C. M., Anderson, C. B., Gesteland, R. F., Atkins, J. F., and Howard, M. T. (2005). Programmed ribosomal frameshifting in decoding the sars-cov genome. *Virology*, **332**(2), 498–510.

Consortium, M. I. *et al.* (2016). The metagenomics and metadesign of the subways and urban biomes (metasub) international consortium inaugural meeting report.

Federhen, S. (2012). The ncbi taxonomy database. *Nucleic acids research*, **40**(D1), D136–D143.

Fisher, P. K. (2020). Sub-biocrust soil microbial communities from mojave desert, california, united states - 8hms. Sequence Read Archive (SRA) [Internet]. Bethesda (MD): National Library of Medicine (US), National Center for Biotechnology Information; 2009. submitted to JGI at 2019-09-20; Available from: https://www.ncbi.nlm.nih.gov/sra/.

Frazee, A. C., Jaffe, A. E., Langmead, B., and Leek, J. T. (2015). Polyester: simulating rna-seq datasets with differential transcript expression. *Bioinformatics*, **31**(17), 2778–2784.

Gevers, D., Knight, R., Petrosino, J. F., Huang, K., McGuire, A. L., Birren, B. W., Nelson, K. E., White, O., Methé, B. A., and Huttenhower, C. (2012). The human microbiome project: a community resource for the healthy human microbiome. *PLoS Biol*, **10**(8), e1001377.

Holtgrewe, M. (2010). Mason: a read simulator for second generation sequencing data.

Li, B. and Dewey, C. N. (2011). RSEM: accurate transcript quantification from RNA-Seq data with or without a reference genome. *BMC Bioinformatics*, **12**(1), 323.

Lonsdale, J., Thomas, J., Salvatore, M., Phillips, R., Lo, E., Shad, S., Hasz, R., Walters, G., Garcia, F., Young, N., *et al.* (2013). The genotype-tissue expression (gtex) project. *Nature genetics*, **45**(6), 580.

Ma, L., Li, B., and Zhang, T. (2014). Abundant rifampin resistance genes and significant correlations of antibiotic resistance genes and plasmids in various environments revealed by metagenomic analysis. *Applied microbiology and biotechnology*, **98**(11), 5195–5204.

Sarkar, H., Zakeri, M., Malik, L., and Patro, R. (2018). Towards selective-alignment: Bridging the accuracy gap between alignment-based and alignment-free transcript quantification. In *Proceedings of the 2018 ACM International Conference on Bioinformatics, Computational Biology, and Health Informatics*, pages 27–36, Washington DC, USA. ACM.

Šošić, M. and Šikić, M. (2017). Edlib: a C/C++ library for fast, exact sequence alignment using edit distance. *Bioinformatics*, **33**(9), 1394–1395.

Srivastava, A., Malik, L., Sarkar, H., Zakeri, M., Almodaresi, F., Soneson, C., Love, M. I., Kingsford, C., and Patro, R. (2020). Alignment and mapping methodology influence transcript abundance estimation. *Genome Biology*, **21**(1), 1–29.

Tang, X., Wu, C., Li, X., Song, Y., Yao, X., Wu, X., Duan, Y., Zhang, H., Wang, Y., Qian, Z., *et al.* (2020). On the origin and continuing evolution of sars-cov-2. *National Science Review*.

Wang, Y., Wang, Y., Chen, Y., and Qin, Q. (2020). Unique epidemiological and clinical features of the emerging 2019 novel coronavirus pneumonia (covid-19) implicate special control measures. *Journal of medical virology*, **92**(6), 568–576.

Wu, F., Zhao, S., Yu, B., Chen, Y.-M., Wang, W., Song, Z.-G., Hu, Y., Tao, Z.-W., Tian, J.-H., Pei, Y.-Y., *et al.* (2020). A new coronavirus associated with human respiratory disease in china. *Nature*, **579**(7798), 265–269.

Zhang, T., Wu, Q., and Zhang, Z. (2020). Probable pangolin origin of sars-cov-2 associated with the covid-19 outbreak. *Current Biology*.
